# Structures of *Streptococcus pyogenes* class A sortase in complex with substrate and product mimics provide key details of target recognition

**DOI:** 10.1016/j.jbc.2022.102446

**Published:** 2022-08-31

**Authors:** D. Alex Johnson, Isabel M. Piper, Brandon A. Vogel, Sophie N. Jackson, Justin E. Svendsen, Hanna M. Kodama, Darren E. Lee, Katy M. Lindblom, James McCarty, John M. Antos, Jeanine F. Amacher

**Affiliations:** Department of Chemistry, Western Washington University, Bellingham, Washington, USA

**Keywords:** sortases, enzymes, target selectivity, structural biology, protein engineering, Abz, 2-aminobenzoyl, baSrtA, *Bacillus anthracis* class A sortase, CWSS, cell wall sorting signal, ESI, electrospray ionization, LII, lipid II, LPXTG, Leu-Pro-X-Thr-Gly, MD, molecular dynamics, MS, spectrometry, MurNAc, muramic acid, MW, molecular weight, NSF, National Science Foundation, PDB, Protein Data Bank, RMSF, root mean square fluctuation, saSrtA, *Staphylococcus* *aureus* class A sortase, SEC, size-exclusion chromatography, SML, sortase-mediated ligation, spySrtA, *Streptococcus pyogenes* class A sortase

## Abstract

The cell wall is a critical extracellular barrier for bacteria and many other organisms. In bacteria, this structural layer consists of peptidoglycan, which maintains cell shape and structural integrity and provides a scaffold for displaying various protein factors. To attach proteins to the cell wall, Gram-positive bacteria utilize sortase enzymes, which are cysteine transpeptidases that recognize and cleave a specific sorting signal, followed by ligation of the sorting signal–containing protein to the peptidoglycan precursor lipid II (LII). This mechanism is the subject of considerable interest as a target for therapeutic intervention and as a tool for protein engineering, where sortases have enabled sortase-mediated ligation or *sortagging* strategies. Despite these uses, there remains an incomplete understanding of the stereochemistry of substrate recognition and ligation product formation. Here, we solved the first structures of sortase A from *Streptococcus pyogenes* bound to two substrate sequences, LPATA and LPATS. In addition, we synthesized a mimetic of the product of sortase-mediated ligation involving LII (LPAT-LII) and solved the complex structure in two ligand conformations. These structures were further used as the basis for molecular dynamics simulations to probe sortase A-ligand dynamics and to construct a model of the acyl–enzyme intermediate, thus providing a structural view of multiple key states in the catalytic mechanism. Overall, this structural information provides new insights into the recognition of the sortase substrate motif and LII ligation partner and will support the continued development of sortases for protein engineering applications.

Bacterial sortases are cysteine transpeptidase enzymes that play important roles at the cell wall of Gram-positive bacteria. Despite over 20 years since the discovery of the first sortase enzyme in *Staphylococcus aureus*, a complete picture of how these critical enzymes recognize their ligands has remained elusive because of limited structural information involving sortases in complex with their substrates (1, 2, 3). This type of characterization is essential to understanding how sortases perform their role of attaching protein factors to the bacterial cell wall (4). A thorough understanding of this process is also relevant to human health and disease in two significant ways; sortases are used in protein engineering, for example, sortase-mediated ligation (SML), *sortagging*, or *sortylation* applications, and sortases are also therapeutic targets for the development of antibiotics (5, 6).

Sortases are widespread in Gram-positive bacteria and are currently grouped into multiple classes (A–F), including several that are considered general housekeeping enzymes (*e.g.*, classes A and E), and those that assemble pili (class C) (4). The sortase mechanism involves two catalytic steps: (i) recognition and cleavage of a target sequence, and formation of an acyl–enzyme, followed by (ii) nucleophilic attack by a second reactant, initiating a ligation reaction that creates a new peptide bond or isopeptide in the case of the bacterial pilus (7, 8). For class A sortases, the general consensus sequence, which is found within the cell wall sorting signal (CWSS), includes a pentapeptide motif, Leu-Pro-X-Thr-Gly (or LPXTG), where X = any amino acid (4). Positions are defined with respect to the location of the cleavage site between the threonine and glycine residues, with P1’ = Gly, P1 = Thr, P2 = X, P3 = Pro, and P4 = Leu (9). For protein anchoring to the bacterial cell surface, the nucleophile in the second step of the reaction mechanism is the cell-wall precursor lipid II (LII), thus allowing for incorporation of the protein into the growing peptidoglycan layer (10).

The majority of knowledge to date on sortase structure and mechanism is focused on class A sortases; however, there are available structures of representative sortases from all six classes (A–F), for example, class A (Protein Data Bank [PDB] ID: 2KID), class B (PDB ID: 1NG5), class C (PDB ID: 3O0P), class D (PDB ID: 2LN7), class E (PDB ID: 5CUW), and class F (PDB ID: 5UUS) (9). These structures have revealed that sortases share a conserved core antiparallel eight-stranded β-barrel structure, termed the *sortase fold* (8, 11). This was first identified in the *S. aureus* class A sortase (saSrtA) structure and is consistently found in wildtype and chimeric sortase enzymes (9, 11, 12). As of early 2022, there were over 65 structures of sortases in the PDB, including from all six classes and SrtA structures from 10 different organisms. Despite this, there is a notable lack of structural information about ligand recognition in sortases. Of the three SrtA structures that contain ligands, two approximate the acyl–enzyme intermediates of saSrtA and *Bacillus anthracis* SrtA (baSrtA) using cleverly designed peptidomimetic ligands (PDB IDs: 2KID and 2RUI). However, because it is not present, these structures do not provide information about recognition of the P1′ residue, a position for which SrtA enzymes have shown variable selectivity *in vitro* (9, 13, 14, 15). The third structure contains a complex between saSrtA and a noncovalently bound LPETG peptide that is shifted by several Angstroms in the peptide-binding pocket (PDB ID: 1T2W), revealing a geometry that is not consistent with known biochemical data (16).

In this work, we have sought to fill remaining gaps in the understanding of SrtA target recognition through the structural characterization of multiple states in the catalytic mechanism of *Streptococcus pyogenes* class A sortase (spySrtA) (Fig. 1). The apo structure of spySrtA was solved using X-ray crystallography in 2009, and its catalytic triad consists of His142, Cys208, and Arg216 (17). Using similar crystallization conditions, we were able to crystallize and solve the structures of a catalytically inactive C208A spySrtA mutant bound to the peptides LPATA and LPATS, which are sequences that are known to serve as spySrtA substrates *in vitro* (17, 18, 19, 20, 21, 22, 23). In addition, we synthesized a model peptide (LPAT-LII) of the ligation product between the LPAT fragment and the *in vivo* nucleophile LII and solved the structures of two complexes between C208A spySrtA and LPAT-LII where the peptide is in the “Thr-in” and “Thr-out” conformations, terminology previously used to describe the side chain of the P1 Thr as protein interacting (“Thr-in”) or solvent interacting (“Thr-out”) (8).

**Figure 1:**
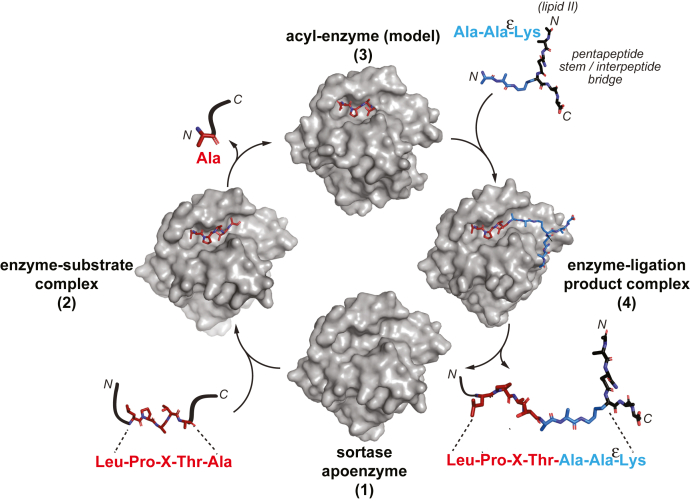
**Structural model of the spySrtA catalytic mechanism.** A summary of the spySrtA catalytic mechanism, as supported by biochemical and structural data in the field, including studies presented here. A portion of the lipid II ligation partner from *Streptococcus pyogenes* is shown, and the structure of this component varies between bacterial species. In this model, LPXTA is shown as the target sequence in the first step of the reaction to reflect the ability of spySrtA to recognize a P1′ Ala residue *in vitro* and to be consistent with the structural data described in this work. The sortase apoenzyme in state 1 is PDB ID: 3FN5. The structures of the enzyme–substrate complex (state 2) and enzyme–ligation product complex (state 4) are the experimental structures presented in this study. As discussed in the main text, the acyl–enzyme (state 3) intermediate is a model generated from experimentally determined structures of the enzyme–substrate complex. PDB, Protein Data Bank; spySrtA, *Streptococcus pyogenes* class A sortase.

Because these are the first solved peptide-bound sortase structures that include the P1′ residue and initial cleavage site, we wanted to investigate the relative dynamics of ligand binding. We ran 900 ns molecular dynamics (MD) simulations using four structures (apo spySrtA [PDB ID: 3FN5]), spySrtA–LPATA, spySrtA–LPATS, and spySrtA–LPAT-LII) to assess positional flexibility and the overall dynamics of the sortase–peptide complex. Finally, we used our peptide-bound structures to model the acyl–enzyme intermediate of spySrtA–LPAT. Taken together, this work provides new structural insights for important states in the SrtA catalytic mechanism (Fig. 1), significantly increasing our understanding of target recognition in this important protein family.

## Results

### Peptide-bound spySrtA crystallization and structure determination

Like other class A sortases, the majority of predicted and verified *in vivo* targets of spySrtA possess LPXTG substrate sequences (24, 25). In addition, prior work from ourselves and others has demonstrated that spySrtA readily accepts LPXTA and LPXTS substrates *in vitro*, despite the fact that these particular sequence variants do not appear to be present in naturally occurring spySrtA substrates *in vivo* (20, 23, 26). The spySrtA enzyme also accepts alanine- or serine-based nucleophiles, which is a characteristic that has been exploited for dual-labeling SML strategies and is consistent with the presence of N-terminal alanines in the interpeptide bridge of LII in *S. pyogenes* (17, 18, 19, 20, 21, 22, 27). Notably, the ability of spySrtA to recognize nonglycine nucleophiles and to accept substrates that vary at the P1′ position is in stark contrast to saSrtA, which is narrowly selective for glycine at these sites (15, 27, 28).

**Figure 2:**
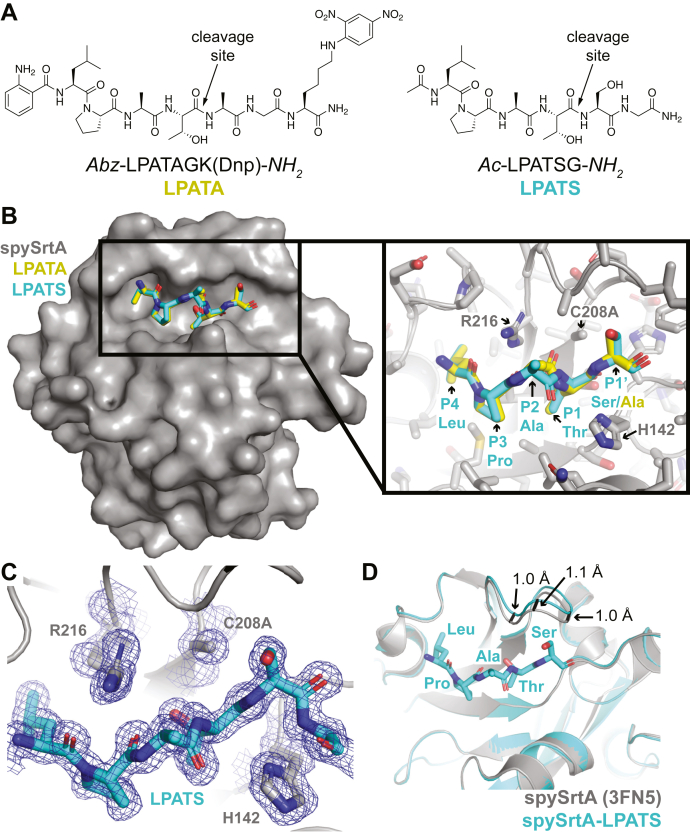
**The spySrtA complex structures with LPATS and LPATA peptides.***A*, structures of model peptides cocrystallized with spySrtA. *B*, the spySrtA protein is in *gray* surface representation, and the noncovalently bound LPATS and LPATA peptides are in *sticks* and colored by heteroatom (N = *blue*, O = *red*, and *C* = *yellow*/*cyan* as labeled). The N-terminal Abz moiety on the LPATA peptide and the P2′ Gly residue for both peptides are not shown in order to focus on the target recognition sequences. The *inset box* shows a zoomed-in version of peptide binding, with all peptide positions labeled. The spySrtA side chains are shown as *sticks* and colored by heteroatom. The catalytic triad (H142–C208A–R216) are labeled. *C*, The 2*F*_o_–*F*_c_ electron density map for the LPATS peptide and catalytic triad is shown in *blue mesh* and rendered at 1.0 σ. The structure is shown as in the *inset* of (*B*). *D*, alignment of the the apo spySrtA (PDB ID: 3FN5) and spySrtA–LPATS structures reveals an RMSD = 0.158 Å (508 main-chain atoms). The proteins are shown in *cartoon* representation and colored as labeled. The LPATS peptide is shown as *sticks* and colored by heteroatom. Abz, 2-aminobenzoyl; PDB, Protein Data Bank; spySrtA, *Streptococcus pyogenes* class A sortase.

In order to gain a stereochemical understanding of target recognition by spySrtA and other class A sortases, we sought to cocrystallize a catalytically inactive mutant (C208A) of spySrtA with a range of model peptides containing known substrate sequences (LPATG/S/A). Briefly, spySrtA protein containing the inactivating C208A mutation was expressed and purified as previously described for the wildtype protein and analyzed *via* SDS-PAGE and LC–electrospray ionization (ESI)–mass spectrometry (MS) (Fig. S1) (23). Purified protein (at ∼1.1 mM) was incubated in a 1:1 ratio with 1 mM peptide for 1 h prior to crystallization by hanging drop vapor diffusion method. Crystallization conditions were optimized from those used for apo spySrtA (PDB ID: 3FN5) and are described in the Experimental procedures section (17). From this, we succeeded in crystallizing and solving two structures of C208A spySrtA bound to the model peptides (P1′ position in **bold**) *Abz*-LPAT**A**GK(Dnp)-*NH*_*2*_ and *Ac*-LPAT**S**G-*NH*_*2*_ (Abz, 2-aminobenzoyl; Fig. 2*A*). The former is an example of a FRET quencher probe that is commonly used for monitoring sortase enzymatic activity (9, 15, 23, 29, 30, 31), whereas the latter is a simplified target containing an acetyl (*Ac*-) cap and C-terminal primary amide (-*NH*_*2*_). For both substrates, LC–ESI–MS was used to confirm that they were cleaved by wildtype spySrtA in a model transacylation reaction (Fig. S2). Notably, we also crystallized C208A spySrtA with peptides containing the canonical LPXTG sequence (*Abz*-LPATGGK(Dnp)-*NH*_*2*_ and fluorescently labeled *5*-*FAM-Ahx*-LPATGG-*NH*_*2*_); however, the crystals obtained were not of suitable diffraction quality.

For simplicity, we will hereafter refer to the solved enzyme–substrate complexes as spySrtA–LPATA and spySrtA–LPATS (Fig. 2*B*). All diffraction and refinement statistics for these complexes are provided in Table 1. In general, crystals grew stacked and were relatively unstable in traditional cryosolutions (*e.g.*, with 10–20% [w/v] glycerol added). As a likely result of these challenges, the crystal ultimately used for spySrtA–LPATA structure determination contained pseudosymmetry. We predict that this may be due to lattice disruption during crystal harvesting. The space group of this crystal was *P* 2_1_ 2_1_ 2_1_ and contained two protomers in the asymmetric unit. We refined it to a *R*_work_/*R*_free_ = 0.21/0.24 at 1.4 Å resolution (Table 1). Relatively high *R*-factors are a consequence of pseudosymmetry in crystal packing (32). Optimization of cryo conditions, namely using PEG 400 as a cryoprotectant, resulted in better quality diffraction data for the crystal used to solve the spySrtA–LPATS structure, as described in the Experimental procedures section. This crystal diffracted to 1.4 Å resolution, and the resulting structure was solved in space group *P* 2_1_ to a *R*_work_/*R*_free_ = 0.17/0.19, with two spySrtA molecules in the asymmetric unit (Table 1). The unit cell and space group are very similar between spySrtA–LPATS and apo spySrtA (17).

**Table 1:** Data collection and refinement statistics

Data collection	spySrtA–LPATA	spySrtA–LPATS	spySrtA-LII “Thr-in”	spySrtA-LII “Thr-out”
Space group	*P* 2_1_ 2_1_ 2_1_ (19)	*P* 2_1_ (4)	*P* 2_1_ 2_1_ 2_1_ (19)	*P* 2_1_ 2_1_ 2_1_ (19)
Unit cell dimensions				
*a*, *b*, *c* (Å)	58.98, 64.57, 75.02	38.49, 59.1, 64.57	34.3, 57.73, 72.25	34.32, 57.68, 71.46
α, β, γ (°)	90, 90, 90	90, 101.7, 90	90, 90, 90	90, 90, 90
Resolution (Å)^a^	48.9–1.4 (1.5–1.4)	43.1–1.4 (1.5–1.4)	45.1–1.8 (1.91–1.8)	44.9–1.9 (2.02–1.9)
*R*_sym_ (%)^b^	8.1 (40.8)	6.1 (31.5)	7.4 (53.0)	8.3 (54.7)
I/σ_I_^c^	12.60 (4.34)	16.86 (4.56)	15.55 (2.47)	16.29 (3.49)
Completeness (%)	99.9 (99.6)	99.6 (98.5)	98.9 (94.0)	98.7 (97.4)
Refinement				

Total no. of reflections	56,989	55,864	13,739	11,555
Reflections in the test set	2838	2709	684	534
*R*_work_^d^/*R*_free_^e^	21.3/23.8	16.9/19.4	17.5/20.6	18.4/22.9
Number of atoms				
Protein	2684	2683	1303	1311
Water	257	449	129	71
Ramachandran plot (%)^f^	99.12/0.88/0	99.42/0.58/0	100/0/0	98.73/1.27/0
*B*_av_ (Å^2^)				
Protein	14.4	14.6	24.3	27.4
Bond length RMSD (Å)	0.007	0.006	0.006	0.007
Bond angle RMSD (°)	0.988	0.872	1.135	1.338
PDB code	7S51	7S40	7T8Y	7T8Z

a Values in parentheses are for data in the highest-resolution shell.

b *R*_sym_ = Σ_*h*_Σ_*i*_ |I(*h*) - I_*i*_(*h*)|/Σ_*h*_Σ_*i*_ I_*i*_(*h*), where I_*i*_(*h*) and I(*h*) values are the *i-*th and mean measurements of the intensity of reflection *h*.

c SigAno = |F(+) - F(−)|/σ.

d *R*_work_ = Σ||F_obs_|_*h*_ - |F_calc_||_*h*_/Σ|F_obs_|_*h*_, *h* ε {working set}.

e *R*_free_ is calculated as *R*_work_ for the reflections *h* ε {test set}.

f Favored/allowed/outliers.

Alignment of chain (or protomer) A of spySrtA–LPATS with the two molecules of spySrtA–LPATA revealed very similar structures, with pairwise RMSD values for main-chain atoms of all protomers of both structures <0.13 Å (Fig. S3*A*). We were able to model all residues of the enzyme in protomer B of spySrtA–LPATA and protomer A of spySrtA–LPATS revealing an additional N-terminal helix not previously seen in the apo structure (Fig. S3*B*). Because of the large degree of similarity between these structures, unless otherwise noted, our analyses will focus on spySrtA–LPATS protomer A.

### Stereochemistry of target recognition by spySrtA

We next used our peptide-bound crystal structures to analyze the stereochemistry of target recognition by class A sortases. In both structures, we see clear peptide density and modeled the entire pentapeptide motif for all spySrtA protomers (Fig. 2, *B* and *C*). Unbiased electron density maps, created by omitting the peptide atoms and running a round of refinement, confirm strong electron density for peptide residues (Fig. S3*C*). Alignment of spySrtA–LPATS with the two (A and B) protomers of apo spySrtA revealed RMSD values for main-chain atoms of 0.158 Å (508 atoms) and 0.189 Å (541 atoms), respectively. The largest difference between these structures is an approximately 1 Å displacement in the backbone of the β7–β8^+3^, β7–β8^+4^, and β7–β8^+5^ loop residues (Fig. 2*D*). Here, superscript numbering refers to the residue position with respect to the catalytic C208 residue, as previously defined (9). This suggests that very small structural rearrangements are needed in order to accommodate the target peptide.

We were able to model the Abz moieties in the spySrtA–LPATA protomers, although the 2,4-dinitrophenyl lysine residue (K(Dnp)) was unresolved. In the A-protomer of spySrtA–LPATA, we see a potential hydrogen bond between the 2-amino group of Abz and the carbonyl of P188 (Fig. S4*A*). While interesting, we do not consider this interaction to be critical for the binding of this substrate, as it is not observed in the B-protomer of the spySrtA–LPATA complex. This is further supported by the successful binding and cocrystallization of the *Ac*-LPATSG-*NH*_*2*_ peptide, which lacks the Abz unit.

**Figure 3:**
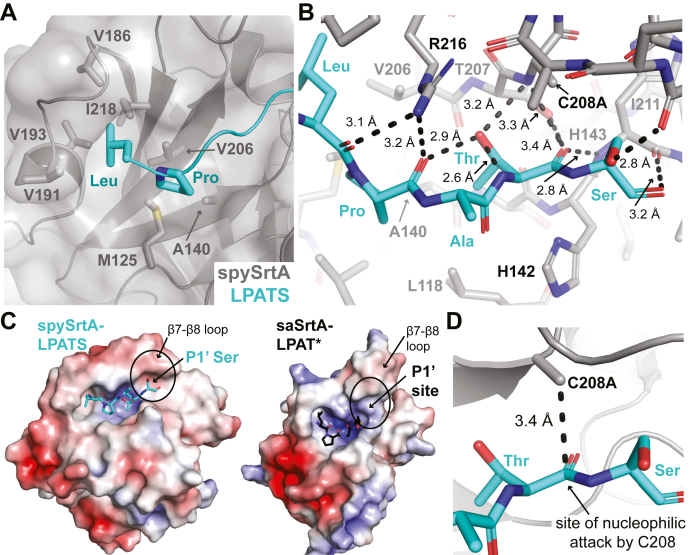
**Stereochemistry of the spySrtA–LPATS interaction.***A,* the interactions of the P4 Leu and P3 Pro ligand residues with spySrtA are highlighted. The spySrtA enzyme is in cartoon and surface representation, with residues that interact directly with the Leu-Pro shown as sticks and colored by heteroatom (C = *gray*, S = *golden yellow*). The ligand is shown as a *cyan cartoon*, with the side chain sticks of Leu-Pro shown and colored by heteroatom (C = *cyan*, N = *blue*). *B,* there are several noncovalent interactions between the LPATS ligand and spySrtA enzyme, shown as *black dashed lines* with distances labeled. There are also intramolecular interactions between the P1 Thr sidechain and its own amide, as well as the P3 Pro carbonyl oxygen, as labeled. The ligand is in stick representation and colored by heteroatom (C = *cyan*, N = *blue*, O = *red*). The spySrtA enzyme is shown as sticks and colored by heteroatom. The catalytic triad (H142-C208A-R216) is labeled. *C,* the electrostatic potential surface maps for SrtA in spySrtA-LPATS (left) and saSrtA-LPAT∗ (right, PDB ID 2KID) were created using APBS in PyMOL and are shown from ± 5 eV, with *red* = negative and *blue* = positive. The ligands are shown as sticks and colored by heteroatom **(**as in *A-B*). The location (spySrtA, left) or predicted location (saSrtA, right) of the P1’ site is circled, and the β7-β8 loop is labeled. *D*, the distance between the CB atom of C208A and the C of the P1 Thr is shown as *black dashed lines* and labeled. This is the site of nucleophilic attack by C208. The structures are rendered as in *B*, with the exception that spySrtA is in cartoon representation with only the side chain sticks of C208A shown.

We next analyzed position-specific interactions in the LPATX motif of the CWSS. The highly conserved Leu residue at P4 interacts with a hydrophobic pocket formed by V186, V191, and V193 of the β6–β7 loop, as well as V206 in β7 and I218 in β8 (Fig. 3*A*). A similar pocket was previously identified in the NMR structure of saSrtA with a covalent peptidomimetic (LPAT∗), PDB ID: 2KID (14). The proline residue in P3 interacts weakly *via* van der Waals interactions with V206 and A140, residues in the β4 and β7 strands, as well as M125 in the β3–β4 loop (Fig. 3*A*). The distances between these residues are of equal magnitude or shorter to those seen in the saSrtA–LPAT∗ structure, where strong intermolecular NOEs were observed that supported P3 Pro interactions with residues in the β4 and β7 strands (Fig. S4*B*) (14).

There are several backbone atoms in the LPATX motif that form noncovalent interactions with residues in spySrtA (Fig. 3*B*). In both the LPATA and LPATS structures, the carbonyl oxygens of the P4 Leu and P3 Pro residues are hydrogen bonded with nitrogen atoms in R216, the catalytic arginine residue. In the LPATA complex, R216 also interacts with the P2 Ala carbonyl, whereas in the LPATS structure, this carbonyl is rotated ∼180º and interacting with solvent (Fig. 3*B*). In all structures, the orientation of the P2 and P1 residue side chains (AT, respectively) observed is rotated ∼180º as compared with the saSrtA–LPAT∗ structure, agreeing more closely with the structure of baSrtA–LPAT∗ from the same group (Fig. S4*C*) (13, 14). As described previously, the conformation observed in spySrtA–LPATA and spySrtA–LPATS is referred to as “Thr-in” to describe the P1 Thr side chain oriented toward the enzyme (8). The carbonyl oxygen of P1 Thr further interacts with the amide of C208A and side chain hydroxyl of T207 as well as the amide of H143, the residue immediately C terminal to the catalytic histidine, H142 (Fig. 3*B*). The methyl group of the P1 Thr is oriented toward the side chain atoms of A140 and V206, and the side chain hydroxyl interacts with the amide of the catalytic C208A residue, as well as forms intrapeptide hydrogen bonds with its own amide and the carbonyl of the P3 Pro (Fig. 3*B*).

Finally, the P1′ Ser in spySrtA–LPATS interacts with a weakly negative ridge formed by the β7–β8 loop, specifically because of E212, the β7–β8^+4^ residue (Fig. 3*C*). A spatially analogous P1′ binding site, albeit with some differences in morphology and overall charge, was predicted in the previously reported saSrtA–LPAT∗ structure (PDB ID: 2KID) (Fig. 3*C*). In our spySrtA–LPATS structure, we also observe a hydrogen bond with the hydroxyl group of the P1′ Ser and the carbonyl of I211 (Fig. 3*B*). This interaction is necessarily absent from the spySrtA–LPATA complex, and therefore, we do not consider it a requirement for substrate binding. In general, the binding site for the P1′ position in spySrtA does not appear to be particularly selective, which is consistent with our previous work on *S. pneumoniae* SrtA (9, 15). Because of the observed similarities in these *Streptococcus* SrtA proteins, as well as our previous work investigating the β7–β8 loop in these proteins, we hypothesize that spySrtA is also nonselective at this position and can accommodate a wide variety of P1′ amino acids (9, 23).

Overall, the observed location for the P1′ Ser, as well as the adjacent P1 Thr, renders the LPATS peptide ideally positioned for nucleophilic attack by the catalytic cysteine residue. Specifically, the methyl group of C208A in the spySrtA–LPATS structure is 3.4 Å from the P1 Thr carbonyl carbon (the corresponding distance in spySrtA–LPATA is 3.2 Å) (Fig. 3*D*). The scissile amide bond of the P1–P1′ linkage is also held in close proximity to the catalytic histidine (His142), which is consistent with the suggested role of this residue in facilitating proton transfers to the excised P1′ fragment and from the incoming LII nucleophile (8). Taken together, these observations support the validity of the spySrtA–LPATS and spySrtA–LPATA complexes as reasonable models for target recognition by class A sortases that are consistent with the current understanding of the sortase catalytic mechanism (4, 8, 33, 34).

### Model of the acyl–enzyme intermediate

**Figure 4:**
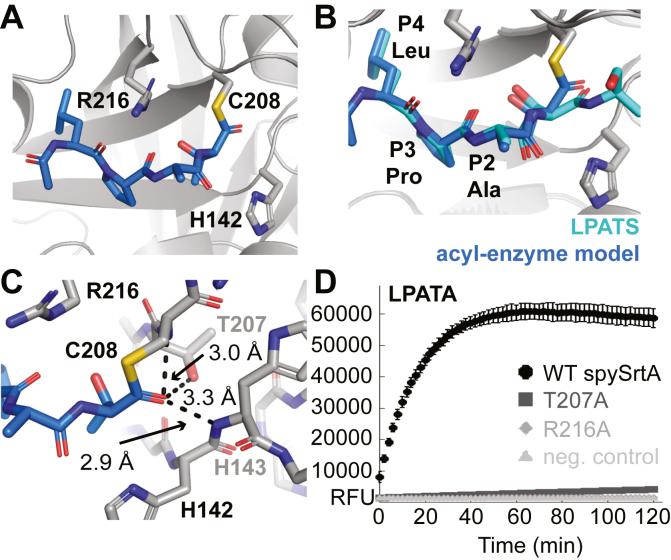
**A model of the acyl–enzyme intermediate of spySrtA–LPAT**. *A*–*C*, the spySrtA protein for the acyl–enzyme intermediate model is in *gray cartoon* (*A* and *B*) or *stick* representation (*C*), with catalytic residue side chains (H142, C208, and R216) and other relevant positions shown as *sticks*, colored by heteroatom (C = *blue/cyan*, O = *red*, N = *blue*, and S = *golden yellow*), and labeled. The spySrtA protein from the spySrtA–LPATS structure (*B*) is rendered similarly but is *darker gray*. The peptides are in *stick* representation, with carbons colored as labeled. *C*, predicted interactions between the P1 Thr carbonyl and spySrtA T207 side chain, C208A amide, and H143 amide are shown as *dashed black lines*, with distances labeled. *D*, triplicate fluorescence data (in relative fluorescence units, RFU) for the reaction of *Abz*-LPATAGK(Dnp)-*NH*_*2*_ and H_2_NOH in the presence of WT (*black circles*), T207A (*dark gray squares*), and R216A (*gray diamonds*) spySrtA protein, and a no protein control (*light gray triangles*). Data for the *Abz*-LPATGGK(Dnp)-*NH*_*2*_ and *Abz*-LPATSGK(Dnp)-*NH*_*2*_ peptides are in Fig. S4*E*. Abz, 2-aminobenzoyl; spySrtA, *Streptococcus pyogenes* class A sortase.

Next, we used our spySrtA–LPATS complex structure to model the acyl–enzyme intermediate (Fig. 4*A*). The model was constructed as described in the Experimental procedures section. Briefly, coordinates for the cleaved peptide were determined and fit into the experimental electron density for spySrtA–LPATS. In addition, C208A was mutated *in silico* to the wildtype cysteine, and a round of refinement was run to validate the peptide geometry. We then performed a steepest descent energy minimization of the acyl–enzyme model to obtain the final geometry (Fig. S8*D*). The resulting acyl–enzyme model is therefore very similar to the spySrtA–LPATS structure, including nearly identical positions for the P4–P2 residues of the LPATS substrate (Fig. 4*B*). Slight differences were observed, however, in the case of the P1 Thr residue. As discussed previously, the P1 Thr carbonyl in spySrtA–LPATS appears to be stabilized by the amides of H143 and C208A as well as the side chain hydroxyl of T207. These interactions were largely maintained in our model; however, a slight rotation of the P1 carbonyl toward T207 was observed (Fig. 4*B*). Specifically, in the geometry of the acyl–enzyme model, the T207 hydroxyl is 3.3 Å from the P1 Thr carbonyl (Fig. 4*C*). This distance is 3.4 Å in the solved structure of spySrtA–LPATS (Fig. 3*B*).

With respect to catalytic mechanism, a feature of the acyl–enzyme model that was also shared by both the spySrtA–LPATS and spySrtA–LPATA structures was the absence of a clear interaction between the P1 Thr carbonyl group and the putative catalytic arginine (R216) side chain. This is significant as this arginine has been proposed to stabilize high-energy oxyanion intermediates generated during the sortase ligation reaction (8, 14, 35). The P1 Thr carbonyl in our acyl–enzyme model and solved structures was actually observed to point away from the R216 side chain, and the distance between these sites is >6 Å (Fig. 4*C*). Nonetheless, R216 was found to be essential for spySrtA function, as mutating it to an Ala residue resulted in complete loss of enzyme activity when tested with model LPATG/S/A peptide substrates (Figs. 4*D* and S4*E*).

In terms of oxyanion stabilization, our structures are more consistent with a key role for the side-chain hydroxyl of T207. This residue, along with the amides of H143 and C208, is ideally positioned to bind to the P1 Thr carbonyl and potentially stabilize tetrahedral oxyanion intermediates formed immediately prior to the acyl enzyme state and following nucleophilic attack by LII (Fig. 4*C*). This type of role for the Thr immediately preceding the catalytic Cys has indeed been suggested in previous computational studies (36). Moreover, sequence analysis of 400 sortase A enzymes in the National Center for Biotechnology Information database reveals that over 90% (363 total) contain a Thr residue immediately preceding the catalytic Cys, which suggests a fundamentally important role for this Thr such as stabilization of key reaction intermediates. Consistent with this hypothesis, we found that a T207A mutant of spySrtA exhibited a near total loss of enzymatic activity (Figs. 4*D* and S4*E*). Notably, a dramatic drop in enzyme activity has also been reported when mutating the corresponding Thr residue (T183) of saSrtA (37).

### Structure and biochemical analyses of spySrtA bound to an LII mimetic

Building from our peptide-bound structures, we next explored the nature of the interaction between spySrtA and its *in vivo* nucleophile, LII. The LII molecule has been identified as the anchor for sortase-catalyzed attachment of many proteins to the bacterial cell wall and serves as a key precursor for the production of peptidoglycan. The nature of this peptidoglycan layer and the cell exterior as a whole is what differentiates Gram-positive and Gram-negative bacteria. Whereas Gram-negative bacteria have an inner membrane surrounded by a relatively thin peptidoglycan layer, followed by a second lipoprotein outer membrane, Gram-positive bacteria lack the outer membrane and contain a relatively thick peptidoglycan layer (38). Although there are exceptions and possible modifications, the main glycan moiety of the peptidoglycan layer consists of alternating GlcNAc and *N*-acetylmuramic acid (MurNAc) residues that are further crosslinked by peptide subunits (38, 39).

The LII building block itself consists of the GlcNAc–MurNAc disaccharide attached to a polyisoprenoid membrane anchor and a pentapeptide stem that is linked *via* an amide bond to the C-3 d-lactyl ether of MurNAc (39). While the structure of the pentapeptide stem varies, a common sequence in Gram-positive bacteria such as *S. pyogenes* is l-alanine, d-isoglutamine, l-lysine, d-alanine, and d-alanine (38, 39, 40). In many of these organisms, the l-lysine is subsequently modified by peptidyltransferases to create an *interpeptide bridge*, which are the residues that ultimately serve as the nucleophile for SML of surface proteins to the peptidoglycan layer. The nature of this interpeptide bridge is variable but commonly includes l-Gly/Ala/Ser residues, for example, for *S. aureus* = Gly_5_, *Enterococcus faecalis* = Ala–Ala, and *Streptococci* = Ala/Ser–Ala (38, 39).

**Figure 5:**
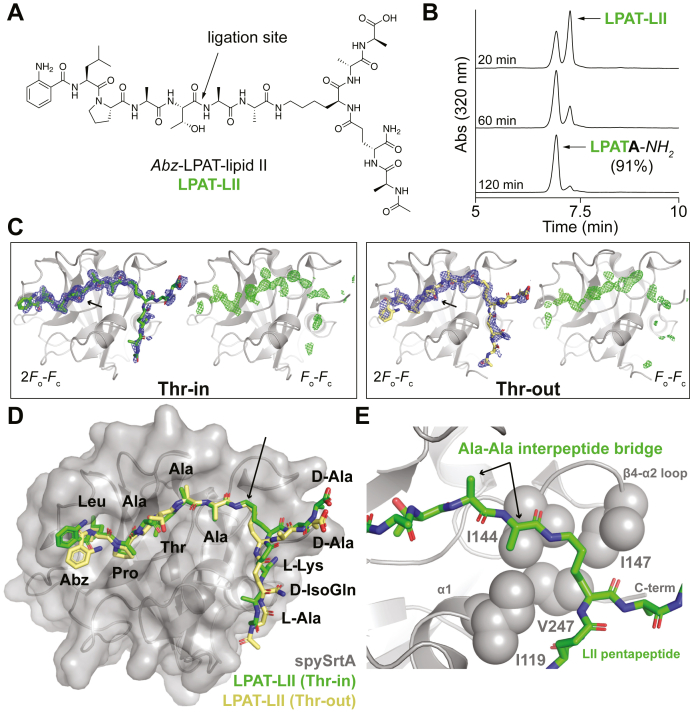
**The structure of spySrtA bound to a peptide model of the LPAT-lipid II ligation product.***A*, chemical structure of the *Abz*-LPAT-lipid II (LPAT-LII) model with the ligation site between the LPAT fragment and lipid II highlighted. *B*, reverse phase-HPLC chromatograms showing efficient cleavage (91% conversion) of the LPAT-LII model in the presence of spySrtA and an excess of alanine amide (A-*NH*_*2*_) nucleophile. Percent conversion was estimated by comparing reverse phase-HPLC peak areas for the unreacted LPAT-II substrate and the LPATA-*NH*_*2*_ product. *C*, the two *F*_o_–*F*_c_ electron density maps for the “Thr-in” and “Thr-out” LPAT-LII peptides are shown at 0.6 σ (*blue mesh*, *left figures*), highlighting the specific, but relatively weak, density in the pentapeptide stem region. The LPAT-LII ligand is in *sticks* and colored as in (*D*). Unbiased *F*_o_–*F*_c_ maps at 2.0 σ are also shown (*green mesh*, *right figures*), which were created by deleting the peptide density and running a round of refinement. The *black arrows* indicate the Thr side chain in both structures. *D*, the structure of spySrtA and the LPAT-LII molecule. This complex was crystallized in both P1 “Thr-in” and “Thr-out” ligand conformations, and both peptides are shown here. SpySrtA is very similar for both structures, and alignment reveals RMSD = 0.082 Å (559 main-chain atoms). Therefore, only the spySrtA protein for the “Thr-in” structure is shown (*gray cartoon* and in surface representation). The LPAT-LII residues are colored as labeled and by heteroatom (N = *blue* and O = *red*). The *black arrow* highlights the Cε atom of the Lys residue of LII, which is the atom at which the conformations of the two solved structures begin to vary, indicating flexibility in the LII pentapeptide. *E*, interactions at the interpeptide bridge of the LPAT-LII ligand are highlighted. Side-chain atoms in spySrtA that form a hydrophobic interaction surface in the vicinity are shown as *spheres*, and the residues are labeled. The “Thr-In” peptide is rendered as in (*D*), and the *arrows* point to the Ala–Ala residues of the interpeptide bridge, as labeled. Abz, 2-aminobenzoyl; spySrtA, *Streptococcus pyogenes* class A sortase.

To visualize the interaction of spySrtA with LII and its related ligation products, we synthesized a model branched peptide representing the ligation of an LPATX substrate to the interpeptide bridge/pentapeptide stem portion of LII from *S. pyogenes* (Fig. 5*A*). Synthesis and characterization are described in the Experimental procedures and Supporting information sections. Specifically, this structure (LPAT-LII) possesses an *Abz*-LPAT fragment derived from the *Abz*-LPATAGK(Dnp)-*NH*_*2*_ substrate described previously covalently linked to an LII mimetic *via* a dialanine interpeptide bridge. To our knowledge, there is no evidence of specific interactions between the glycan residues of LII and the sortase enzyme; therefore, those portions were omitted and replaced with a simple acetyl group on the terminal l-alanine residue. We also note that some structural heterogeneity in the interpeptide bridge/pentapeptide stem of *S. pyogenes* is likely. Examples of this include variable numbers of alanine residues in the interpeptide bridge and even low levels of hydroxylysine (38, 41). However, our LPAT-LII model is consistent with structural features reported in the literature and should therefore be representative of a significant fraction of LII structures in *S. pyogenes* (17, 38, 42).

As a preliminary assessment of whether our LPAT-LII model was recognized by the enzyme, it was used in a model of spySrtA-catalyzed reaction and found to be efficiently cleaved at the expected site between the Thr and Ala residues (Figs. 5*B* and S5). Indeed, we found LPAT-LII to react more rapidly than the related *Abz*-LPATAGK(Dnp)-*NH*_*2*_ peptide, suggesting that the added interpeptide bridge/pentapeptide stem portion may be enhancing binding and recognition by spySrtA (Fig. S5).

We next crystallized and solved the structure of C208A spySrtA noncovalently bound to our LPAT-LII mimetic. Two distinct conformations were observed, with the peptide Thr residue in both the “Thr-in” and “Thr-out” conformations previously observed in other SrtA structures (Fig. 5, *C* and *D*) (13, 14). These structures will be referred to as spySrtA–LPAT-LII “Thr-in” and spySrtA–LPAT-LII “Thr-out.” Crystallization was performed similarly to the peptide-bound structures described previously, and as in the Experimental procedures section. Microseeding was used in this case to obtain crystals of suitable diffraction quality. Both the “Thr-in” and “Thr-out” structures crystallized in the space group *P* 2_1_ 2_1_ 2_1_ with one molecule in the asymmetric unit and to a resolution of 1.8 Å and 1.9 Å, respectively. The spySrtA–LPAT-LII “Thr-in” structure was refined to a final *R*_work_/*R*_free_ = 0.18/0.21 and the spySrtA–LPAT-LII “Thr-out” structure to a final *R*_work_/*R*_free_ = 0.18/0.23 (Table 1). Overall, the structures are very similar, and the main chain atoms of spySrtA align with an RMSD = 0.082 Å (559 atoms).

In the spySrtA–LPAT-LII “Thr-in” structure, the stereochemistry of the LPATA portion is consistent with our peptide-bound structures (Fig. S6*A*). The main-chain atoms align to the A- and B-protomer of spySrtA–LPATA with an RMSD = 0.218 Å (518 atoms) and 0.205 Å (495 atoms), respectively. Values are almost identical for spySrtA–LPAT-LII “Thr-out,” at 0.218 Å (497) and 0.207 Å (489) for the spySrtA–LPATA A- and B-protomers. The positions of the interpeptide bridge dialanine and ε-amine/ε-carbon of the l-lysine residue are also well conserved between the “Thr-in” and “Thr-out” structures (*gray arrow* in Fig. 5*D*). These sites make contacts with residues of the β7–β8 loop and appear to be stabilized by a hydrophobic pocket in spySrtA formed by four amino acids (I119 in α1, I144 and I147 in the β4–α2 loop, and V247 at the C terminus) (Fig. 5*E*). Moving beyond the ε-carbon of l-lysine, there is more variability in the conformation of the pentapeptide stem between the two structures; this reflects the weaker electron density for these residues (Fig. 5, *C* and *D*). Indeed, in both structures, there is only one observed noncovalent interaction with the LII pentapeptide and spySrtA enzyme, a hydrogen bond formed between the spySrtA α1 Y120 hydroxyl and the amide of the LII d-isoglutamine residue (Fig. S6*B*). In each, there are also multiple interactions with the LII pentapeptide and spySrtA enzyme of molecules related by symmetry (Fig. S6*C*).

Taken together, our crystallographic findings suggest that while the interpeptide bridge likely plays an important role in SrtA recognition of LII, the pentapeptide stem does not substantially interact with the enzyme. As noted previously, the electron density for the pentapeptide stem was weaker than that of the LPAT segment and interpeptide bridge dialanine, suggesting flexibility in the stem region of the LPAT-LII ligand (Fig. 5*C*). Nonetheless, the clear electron density for the dialanine interpeptide bridge revealed a discrete binding site with potential implications for substrate binding outside the standard LPXTG substrate motif, specifically at the P2′ position. Interestingly, several predicted *in vivo* substrates of *S. pyogenes* and other streptococcal species possess LPXTGE motifs, with glutamic acid occupying this P2′ position (25). In our hands, preliminary experiments suggest that spySrtA recognizes LPATGG and LPATGE peptides similarly, but additional work is ongoing to investigate P2′ specificity (data not shown).

### MD simulations of spySrtA bound to target peptides

During structure refinement and model building for spySrtA–LPAT-LII, we observed reduced electron density for the pentapeptide stem as compared to the LPAT sequence and interpeptide bridge, which suggested variations in conformational dynamics for different segments of the LPAT-LII ligand (Fig. 5*C*). To probe this further, as well as investigate the MD of our other spySrtA substrate complexes, we ran ∼900 ns MD simulations of apo spySrtA (PDB ID: 3FN5), spySrtA–LPATA, spySrtA–LPATS, and spySrtA-LII “Thr-in” structures (Table S1). Briefly, MD simulations were performed in full atomistic detail with explicit water using the AMBER99SB∗-ILDN force fields (43). The starting structures were solvated with ∼10,000 TIP3P water molecules in a cubic box with periodic boundary conditions. The system was neutralized with an ionic concentration of 150 mM. These simulations are described in further detail in the Experimental procedures and Supporting information sections.

**Figure 6:**
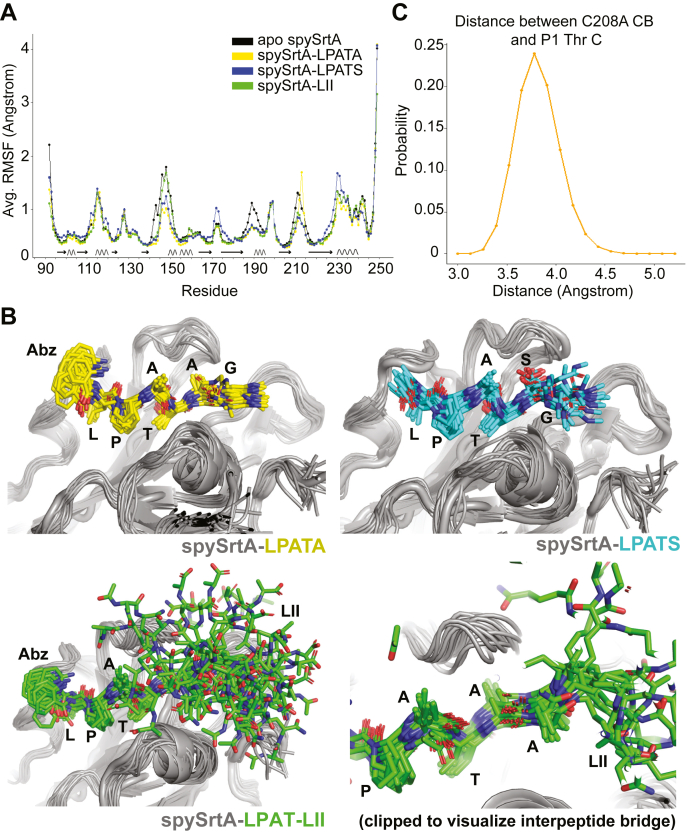
**Molecular dynamics simulations of spySrtA structures**. *A*, the average root mean square fluctuation (RMSF) of backbone atoms in each residue are shown and colored as labeled. The secondary structure elements are indicated under the curve by *arrows* for β-strands and *curved lines* for α-helices. These are based on the apo spySrtA structure (PDB ID: 3FN5), and residue boundaries fluctuate slightly amongst the structures. *B*, representative frames from every approximately 45 ns of simulation time are aligned to the original model for spySrtA–LPATA (*top left*, *yellow peptide*), spySrtA–LPATS (*top right*, *cyan peptide*), and spySrtA–LPAT-LII (*bottom*, *green peptide*) simulations. A clipped version of the spySrtA–LPAT-LII simulation is shown in the *bottom right* to highlight the relative inflexibility of the interpeptide bridge dialanine residues as compared with the rest of the pentapeptide stem. For all images in (*B*), the spySrtA protein is in *gray cartoon*, and the peptides are shown in *stick* representation and colored by heteroatom (N = *blue* and O = *red*). *C*, the distribution of observed distances between the C208A CB (C_β_) and P1 Thr C (main-chain carbonyl carbon) is shown as a function of its probability. The highest probability distance is approximately 3.8 Å. PDB, Protein Data Bank; spySrtA, *Streptococcus pyogenes* class A sortase.

Overall, the RMSD of backbone atom positions for spySrtA indicated that the enzyme remained stable over the course of all four simulations. (Fig. S7*A*). The root mean square fluctuation (RMSF) of backbone atoms for spySrtA were also consistent with a well-defined eight-stranded antiparallel β-barrel sortase core structure, in that these regions are relatively inflexible over the course of the simulation, as compared with some α-helical and all loop regions (Fig. 6*A*). In all the peptide ligands, the LPATX sequences were also relatively inflexible. This was clearly evident in the visualization of representative frames taken over the course of each simulation (Fig. 6*B*) as well as the average RMSF of backbone atoms in each peptide (Fig. S7*B*). The RMSF of the P1′ Ser in the LPATS peptide was also similar to that of the P1′ Ala in either the LPATA or LPAT-LII simulations.

We also analyzed the distance distribution between the C208A methyl group (or C_β_ atom) and that of the P1 Thr carbonyl C in the spySrtA–LPATA simulation, revealing that the most often sampled distance equals 3.8 Å (Fig. 6*C*). Surprisingly, our experimentally observed distance of 3.4 Å (Fig. 3*D*) was observed less than 5% of the time in the simulation; however, considering the C208A mutation and the standard C-S bond length of ∼1.8 Å, this distribution of distances still positions the P1 Thr C in an ideal position for nucleophilic attack by the thiol group of the catalytic cysteine.

Finally, analysis of the average RMSF of every nonhydrogen atom in the LPAT-LII ligand was consistent with increased conformational dynamics for the pentapeptide stem portion (Fig. S7*C*). We see a dramatic increase in flexibility in atoms in the LII pentapeptide, as compared with the LPAT and interpeptide bridge sequences (Fig. S7*C*). Specifically, this increase begins at the Cε atom of the lysine side chain and gets progressively larger moving down the lysine side chain toward the pentapeptide stem. This was also clearly evident in the alignment of representative frames (taken every 45 ns) from the MD trajectory of the spySrtA–LPAT-LII system (Fig. 6*B*). Taken together, these MD simulations strongly support our described structure-based conclusions.

## Discussion

As we highlight in Figure 1, there are multiple key states in the SrtA catalytic cycle when attaching a protein to the cell surface of Gram-positive bacteria. Facilitated by a conserved Cys-His-Arg triad, the apo enzyme (state 1) recognizes a motif within the CWSS on the C terminus of a target protein (state 2) and cleaves the peptide between the P1 Thr and P1′ Gly residues (or other P1′ residues *in vitro*), presumably forming a tetrahedral oxyanion that resolves to generate a thioacyl–enzyme intermediate (state 3). Nucleophilic attack by the N-terminal amine of the interpeptide bridge of LII on the carbonyl carbon of the P1 Thr residue leads to a second tetrahedral oxyanion intermediate that collapses into the final ligation product and completes the transpeptidation reaction, whereby the initial target sequence (minus the P1′ residue and all residues C terminal to this position) is covalently attached to LII (state 4) (8, 10, 44). Using spySrtA as a model, we solved structures that experimentally show how the full LPXTX substrate is recognized by the enzyme (state 2) as well as how the final ligation product is accommodated within the enzyme active site (state 4). In addition, we used our peptide-bound structures and energy minimization to model the acyl–enzyme intermediate (state 3); thus, providing a nearly comprehensive structural view of the spySrtA catalytic mechanism.

Considered alongside other SrtA structures that contain bound substrate mimetics, the studies reported here both reaffirm certain common structural features and reveal new insights. As described previously, we observe several position-specific interactions similar to those first reported for the peptidomimetic-bound structures of saSrtA and baSrtA (13, 14). Our observed interactions at the P4 Leu, P3 Pro, P2 Ala, and P1′ Ala/Ser positions also support additional data on substrate selectivity in class A sortases (9, 15, 27, 45). Finally, in our LPAT-LII structure, we see both “Thr-in” and “Thr-out” conformations, molecular orientations that have also been previously described (8, 13, 14).

However, apart from the orientation of the P1 Thr side chain, other aspects of the positioning of this residue reveal unique attributes of our spySrtA complexes that differ from prior work with baSrtA and saSrtA (13, 14). It was previously suggested that the carbonyl group of the P1 Thr may be stabilized by contacts with the highly conserved Arg residue that forms part of the ubiquitous Cys-His-Arg triad found in sortases. This proposed interaction would further allow the Arg side chain to stabilize tetrahedral oxyanion intermediates generated during the sortase-catalyzed transpeptidation reaction. While we do find that the conserved Arg (R216) of spySrtA is critical for enzyme function (Figs. 4*D* and S4*E*), and appears to play a role in positioning the LPXTX motif through direct contacts with the P4 and P3 carbonyl groups (Fig. 3*B*), we see no evidence for interactions with the P1 carbonyl. Indeed, the P1 carbonyl in our complexes is projected away from the R216 side chain, and instead forms interactions with a series of other sites (Figs. 3*B* and 4*C*), including the hydroxyl group of a conserved Thr residue (T207) adjacent to the active site Cys (C208). These same contacts would also appear to provide a suitable oxyanion hole for stabilizing high-energy reaction intermediates, which is supported by our finding that a T207A mutant of spySrtA was essentially inactive (Figs. 4*D* and S4*E*).

Our observations with the P1 Thr indicate that further work on the exact role of the conserved Arg residue in sortase catalysis is warranted. Along these lines, intriguing results from a recent directed evolution study suggest that the conserved Arg in sortase A enzymes may primarily be responsible for substrate positioning and binding, as opposed to stabilization of catalytic intermediates. Specifically, an engineered variant of saSrtA was reported that is selective for an LMVGG substrate motif (46). Remarkably, in this enzyme, the conserved Arg of wildtype saSrtA was mutated to Ser, and yet it remained an efficient transpeptidase. While it is possible that this highly mutated saSrtA variant acquired a series of compensatory mutations that negated the need for Arg to stabilize high-energy oxyanion intermediates, we would argue an alternate interpretation that the wildtype Arg is not critical for creating an oxyanion hole and rather its primary function is substrate binding and controlling substrate selectivity. The Arg to Ser mutation in the LMVGG-specific saSrtA variant is thus understood as contributing to a change in substrate selectivity as opposed to representing a fundamental change in the catalytic mechanism.

We anticipate that our work will also prove useful in the continued development of SML protein modification strategies. Structure-guided engineering efforts have already seen success in generating sortases with altered substrate selectivity or increased activity, as well as a saSrtA mutant that no longer requires a Ca^2+^ cofactor (9, 47, 48, 49). Moving forward, further optimization of spySrtA and other class A sortases for use in SML can be envisioned based on the molecular characteristics elucidated in the spySrtA complexes and related structures presented here. In addition, the extended target binding cleft revealed in our spySrtA–LPAT-LII structures suggests that portions of the substrate outside the LPXTX motif could make specific contacts with the spySrtA enzyme, for example, residues in the P2′ site. A systematic exploration of how these positions impact enzymatic activity *in vitro* may therefore be helpful in optimizing SML using spySrtA and other class A sortases. A similar approach has already proven beneficial for saSrtA, where it is known that a P2′ Gly residue generally provides superior reactivity *in vitro* (50).

In summary, this work reports the first crystal structures of spySrtA bound to an LPXTX substrate as well as a model of the *in vivo* ligation product involving LII. These structures reveal new details on substrate recognition by bacterial sortases, which may prove valuable for the use of sortases as tools for protein engineering. More broadly, this work improves our understanding of the fundamental enzymology of this large and clinically relevant class of bacterial enzymes.

## Experimental procedures

### Expression and purification of spySrtA protein

Wildtype spySrtA, C208A spySrtA, T207A spySrtA, and R216A spySrtA genes were recombinantly expressed using *Escherichia coli* BL21 (DE3) cells in the pET28a(+) vector (Genscript), as previously described (23). The wildtype sequence used matches that of the published spySrtA structure, PDB ID: 3FN5 (17). Briefly, transformed cells were grown at 37 °C in LB media to an absorbance of 0.6 to 0.8 at 600 nm, followed by induction using 0.15 mM IPTG for 18 to 20 h at 18 °C. The cells were harvested in lysis buffer (0.05 M Tris [pH 7.5], 0.15 M NaCl, and 0.5 mM EDTA], and whole-cell lysate was clarified using centrifugation, followed by filtration of the supernatant. Initial purification was conducted using a 5 ml HisTrap HP column (Cytiva) and wash (0.05 M Tris [pH 7.5], 0.15 M NaCl, 0.02 M imidazole, and 0.001 M Tris(2-carboxyethyl)phosphine) and elution (wash buffer with 0.3 M imidazole) buffers.

Following immobilized metal affinity chromatography, the His tag was proteolyzed off the N terminus of the C208A spySrtA protein using tobacco etch virus protease overnight at 4 °C and a ratio of ∼1:100 (tobacco etch virus:protein). The proteins used for activity assays (wildtype, T207A, and R216A) were not cleaved, consistent with our previous work (9, 23). After collecting the flow-through of a second 5 ml HisTrap HP column (wash buffer identical to that described previously), size-exclusion chromatography (SEC) was conducted using a HiLoad 16/600 Superdex 75 column (Cytiva) in SEC running buffer (0.05 M Tris [pH 7.5], 0.15 M NaCl, and 0.001 M Tris(2-carboxyethyl)phosphine). Purified protein fractions corresponding to the monomeric peak were pooled and concentrated using an Amicon Ultra-15 Centrifugal Filter Unit (10,000 NWML). Protein concentrations were determined using theoretical extinction coefficients calculated using ExPASy ProtParam (Swiss Institute of Bioinformatics; https://web.expasy.org/protparam/) (51). Protein not immediately used was flash frozen in SEC running buffer and stored at −80 °C.

The purity, monomeric state, and identity of purified enzymes were confirmed by SDS-PAGE, analytical SEC, and LC–ESI–MS, respectively. For LC–ESI–MS, analyses were performed on an Agilent 6545XT AdvanceBio Q-TOF system interfaced with an Agilent 1290 HPLC system. Separations upstream of the Q-TOF were achieved with a Phenomenex Aeris 3.6 μM WIDEPORE C4 200 Å column (100 × 2.1 mm) (H_2_O [0.1% formic acid]/MeCN [0.1% formic acid] mobile phase at 0.3 ml/min, method: hold 10% MeCN 0.0 to 1.0 min, linear gradient of 10 to 90% MeCN 1.0 to 9.0 min, hold 90% MeCN 9.0 to 11.0 min, linear gradient of 90 to 10% MeCN 11.0 to 11.1 min, re-equilibrate at 10% MeCN 11.1 to 15.0 min). Deconvolution of protein charge ladders was achieved using Agilent MassHunter BioConfirm software (version 10.0). The expected and observed molecular weights (MWs) for all proteins in this study were as follows: wildtype spySrtA (calculated average MW = 20,657.5 Da, observed = 20,657.6 Da), C208A spySrtA (calculated average MW = 18,573.3 Da, observed = 18,573.4 Da), T207A spySrtA (calculated average MW = 20,627.3 Da, observed = 20,627.5 Da), and R216A spySrtA (calculated average MW = 20,572.3 Da, observed = 20,572.5 Da). Representative MS data for wildtype and C208A spySrtA are also provided in Fig. S1.

### Peptide synthesis

Model peptide substrates used in crystallization and/or enzyme assays with the general structure *Abz*-LPATXGK(Dnp)-*NH*_*2*_ (*Abz* = 2-aminobenzoyl; Dnp = 2,4-dinitrophenyl; *NH*_*2*_ = C-terminal primary amide) were synthesized and purified as previously described (9). The *Ac*-LPATSG-*NH*_*2*_ peptide (*Ac* = acetyl; *NH*_*2*_ = C-terminal primary amide) used for spySrtA–LPATS cocrystallization was purchased from Biomatik. *5*-*FAM-Ahx*-LPATGG-*NH*_*2*_ (5-FAM-Ahx = 5-carboxyfluorescein linked *via* an aminohexanoic acid linker; *NH*_*2*_ = C-terminal primary amide) used in attempted cocrystallization studies was also purchased from Biomatik. Finally, the synthesis of *Abz*-LPAT-LII was achieved *via* manual Fmoc solid phase peptide synthesis. Full experimental details for the preparation of LPAT-LII are provided in the Supporting information and Fig. S8.

### HPLC and LC–MS characterization of spySrtA-catalyzed reactions

LPATS/LPATA/LPAT-LII peptide substrates (50 μM), alanine amide nucleophile (5 mM), and wildtype spySrtA enzyme (5 μM in the reaction with LPATS, otherwise 1 μM) were combined at room temperature and incubated for the times indicated. All reactions contained 10% (v/v) sortase reaction buffer (500 mM Tris [pH 7.5] and 1500 mM NaCl) as well as ≤1.1% (v/v) residual dimethyl sulfoxide from the peptide substrate stock solutions. Reactions were analyzed using a Dionex Ultimate 3000 HPLC system interfaced with an Advion CMS expression^L^ mass spectrometer. Separations were achieved with a Phenomenex Kinetix 2.6 μM C18 100 Å column (100 × 2.1 mm) (aqueous [95% H_2_O, 5% MeCN, 0.1% formic acid]/MeCN [0.1% formic acid] mobile phase at 0.3 ml/min, gradients adjusted for each substrate to achieve separation between relevant reaction components).

### Fluorescence assay for sortase activity

Enzyme assays for assessing the reactivity of wildtype spySrtA *versus* the T207A and R216A mutants were conducted using a Biotek Synergy H1 plate reader as previously described (9). Briefly, *Abz*-LPATXGK(Dnp)-*NH*_*2*_ peptide substrates (50 μM final concentration) were incubated with hydroxylamine nucleophile (5 mM) and sortase enzyme (5 μM) at room temperature. All reactions contained 10% (v/v) 10× sortase reaction buffer (500 mM Tris [pH 7.5] and 1500 mM NaCl) and small amounts of residual dimethyl sulfoxide (≤0.9% v/v) from the peptide stock solutions. The fluorescence intensity of each reaction well was measured at 2 min time intervals over a 2 h period (λ_ex_ = 320 nm; λ_em_ = 420 nm; and detector gain = 75). All reactions were performed in triplicate, and fluorescence intensity (in relative fluorescence units) over time was plotted using Kaleidagraph 5.01 (Synergy software).

### Crystallization of spySrtA complex structures

The C208A spySrtA protein was crystallized at approximately 20 mg/ml or 1.1 mM. Peptide (LPATA, LPATS, or LPAT-LII), at 1 mM final concentration, was incubated with protein in a 1:1 ratio at room temperature for approximately 1 h prior to crystallization by hanging drop vapor diffusion method using a 500 μl well solution to protein solution ratio of 1:1, for a final drop volume of 4 μl (2 μl + 2 μl). Crystallization conditions were optimized using those for the wildtype apo protein (17). The crystallization conditions for the crystals used for data collection were (for all, containing C208A spySrtA): LPATA (0.1 M sodium acetate, 34% [w/v] PEG 8000, 0.1 M Tris [pH 6]), LPATS (0.1 M sodium acetate, 30% [w/v] PEG 8000, 0.1 M Tris [pH 6]), LPAT-LII “Thr-in” (0.15 M sodium acetate, 26% [w/v] PEG 8000, 0.1 M Tris [pH 6]), and LPAT-LII “Thr-out” (same conditions as LPAT-LII “Thr-in”). Microseeding was used to obtain crystals of suitable diffraction quality for structure determination with the LPAT-LII-bound complexes, using initial crystals that grew in conditions of higher PEG 8000 concentration (>30% [w/v], consistent with the other conditions described). As described in the main text, glycerol was used as a cryoprotectant for the C208A spySrtA–LPATA crystal (cryo: crystallization conditions plus 12% [w/v] glycerol), but for the other crystals, PEG 400 was used (cryo: 0.15 M sodium acetate, 10% [w/v] PEG 8000, 40% [w/v] PEG 400, 0.1 M Tris [pH 6]). The crystals were flash cooled by plunging into liquid nitrogen.

### Data collection, structure determination, and protein analyses

Data were collected at the Advanced Light Source at Lawrence Berkeley National Laboratory on beamline 5.0.1 and 5.0.2, at λ= 1.00004 nm or 0.97741 nm over 360°, with Δφ = 0.25° frames and an exposure time of 0.5 s per frame. Data were processed using the XDS package (Max Planck Institute for Medical Research) (Table 1) (52, 53). Molecular replacement was performed using Phenix with spySrtA (PDB ID: 3FN5) used as the search model. Refinement was performed using Phenix (The Phenix Industrial Consortium), manual refinement was done using Coot (MRC Laboratory of Molecular Biology), and model geometry was assessed using MolProbity (Duke University) and the PDB validation server (54, 55, 56). Coordinates for the Abz moiety in C208A spySrtA–LPATA and LPAT-LII were initially determined using phenix.eLBOW from the SMILES (Simplified Molecular Input Line Entry System) strings rendered using ChemDraw (PerkinElmer Informatics) (57). All crystal data and refinement statistics are provided in Table 1. Structural analyses and figure rendering were done using PyMOL (Schrödinger software). PDB accession codes for the structures presented here are provided in Table 1 and are (for all, containing C208A spySrtA): LPATA (PDB ID: 7S51), LPATS (PDB ID: 7S40), LPAT-LII “Thr-in” (PDB ID: 7T8Y), and LPAT-LII “Thr-out” (PDB ID: 7T8Z).

### MD simulations of spySrtA

All MD simulations were performed using GROMACS 2020.4 (GROMACS development teams at the KTH Royal Institute of Technology and Uppsala University) with the AMBER99SB∗-ILDN force fields (43, 58, 59, 60). Additional details and relevant references are in the Supporting information section (61, 62, 63, 64, 65, 66, 67, 68, 69, 70).

For energy minimization of the spySrtA–LPAT model, a steepest descent energy minimization was performed on the solvated system with a maximum force tolerance of 500 kJ/mol/nm. The steepest descent converged in 2998 steps.

## Data availability

All data are contained in the article and the supporting information.

## Supporting information

This article contains supporting information (61, 62, 63, 64, 65, 66, 67, 68, 69, 70).

## Conflict of interest

The authors declare that they have no conflicts of interest with the contents of this article.
