# Supplementary material for: Structures of Streptococcus pyogenes class A sortase in complex with substrate and product mimics provide key details of target recognition
Source: J Biol Chem. 2022 Aug 31;298(10):102446. doi: 10.1016/j.jbc.2022.102446 (PMC9520033; doi:10.1016/j.jbc.2022.102446)
Supplement: Supplemental Data [file mmc1.docx]

**Supplementary Information for**

**Structures of *Streptococcus pyogenes* Class A sortase in complex with substrate and product mimics provide key details of target recognition**

D. Alex Johnson, Isabel M. Piper, Brandon A. Vogel, Sophie N. Jackson, Justin E. Svendsen, Hanna M. Kodama, Darren E. Lee, Katy M. Lindblom, James McCarty, John M. Antos, Jeanine F. Amacher

*Corresponding Authors:

John Antos

**Email:** [antosj@wwu.edu](mailto:antosj@wwu.edu)

Jeanine Amacher

**Email:** [amachej@wwu.edu](mailto:amachej@wwu.edu)

**This PDF file includes:**

Supplementary Methods (pp 2-3)

Figures S1 to S8 (pp 4-11)

Tables S1 (pp 12)

SI References (pp 13)

**Table of Contents**

**Supplemental Methods**  2

**Figure S1.** Sequences and LC-ESI-MS characterization of wild-type and C208A spySrtA. 4

**Figure S2.** Model transacylation reactions of spySrtA with LPATA and LPATS substrates. 5

**Figure S3.** Structural alignments of spySrtA complex structures. 6

**Figure S4.** The spySrtA LPATA- and LPATS-complex structures. 7

**Figure S5.** Comparison of transacylation reactions with LPAT-LII and LPATA 8

**Figure S6.** Structure of spySrtA bound to LPAT-LII peptide. 9

**Figure S7.** Molecular dynamics simulations of spySrtA. 10

**Figure S8.** Synthesis and characterization of LPAT-LII. 11

**Table S1.** Details of the molecular dynamics simulation size. 12

**References**  13

Supplementary Information Text

**Supplemental Methods**

**Molecular dynamics simulations.** Nonstandard residues of the lipid II pentapeptide or LPAT-LII ligand that are not represented by the standard AMBER force field were described by the General Amber Force Field (GAFF2) (1). Partial charges for the ligand atoms were obtained using the restrained electric potential fitting method (RESP) with the molecular electric potentials computed at the HF/6-31G* level of theory (2). Quantum chemistry calculations were performed using GAMESS with RESP fitting performed using Multiwfn (3, 4). The starting protein structures were solvated with TIP3P water molecules in a cubic box with periodic boundary conditions. The system was neutralized with an ionic concentration of 150 mM. The total number of atoms, box dimensions, and simulation time is reported in **Table S1**). Long-range electrostatic interactions were treated with the particle mesh Ewald (PME) algorithm (5). A cutoff of 1.0 nm was used for both the real-space Coulombic and Lennard-Jones interactions. Following a steepest descent energy minimization, a short 100 ps simulation was performed with position restraints on all protein heavy atoms in the NVT ensemble at 300 K using the velocity rescaling thermostat (6). This was followed by a 1 ns equilibration in the NPT ensemble at 1 bar without position restraints using a Parrinello-Rahman barostat (7). We used an integration time step of 2 fs. All bonds to hydrogen atoms were constrained using the LINCS algorithm (8). Production runs of ~900 ns were performed in the NVT ensemble at 300 K. Production simulations were performed on *Expanse*, an NSF-funded system operated by the San Diego Supercomputer Center at UC San Diego, available through the XSEDE program (9). To monitor distances between atoms of interest, we used the PLUMED2 plugin (10).

**Synthesis of LPAT-LII**. **Reagents and General Procedures.** The synthesis of LPAT-LII was achieved via manual Fmoc solid phase peptide synthesis (SPPS) using Fmoc D-Ala Wang resin (AAPPTec) (**Fig. S8A**). Unless noted otherwise, all steps (washing, coupling, deprotection) were performed at room temperature and included gentle agitation on a bench-top rocking platform. Incorporation of D-isoglutamine was achieved using a commercially available building block (Fmoc-D-isoGln-OH) purchased from AAPPTec. The 4-methyltrityl (Mtt) protected lysine residue (Fmoc-Lys(Mtt)-OH) used to create the isopeptide linkage in LPAT-LII was purchased from AAPPTec. Boc-2-aminobenzoic acid for installation of the 2-aminobenzoyl (Abz) fluorophore was obtained from Chem-Impex International. All other materials and reagents were obtained from commercial sources and used without further purification.

**Synthesis.** A 15 mL polypropylene synthesis vessel fitted with appropriate frits and inlet/outlet caps was loaded with 0.286 g (0.2 mmol scale) of Fmoc-D-Ala Wang Resin (**S1**, 0.7 mmol/g). The resin was then swollen prior to synthesis with ~20 mL of N-methyl-2-pyrrolidinone (NMP) (3x, 10 min per wash). Next, the base-labile Fmoc group was removed with 20 mL of 20% piperidine in NMP (2x, 10 min per treatment), followed by washing with ~20 mL of NMP (3x, 5 min per wash). The resin was then elaborated through sequential coupling of Fmoc-D-Ala-OH, Fmoc-Lys(Mtt)-OH, Fmoc-D-isoGln-OH, and Fmoc-Ala-OH. For each residue, a coupling solution consisting of Fmoc amino acid (0.6 mmol), O-(benzotriazol-1-yl)-N,N,N’,N’-tetramethyluronium (HBTU) (0.6 mmol), and N,N-diisopropylethylamine (DIPEA) (1.0 mmol) in ~6 mL of NMP was used. Following thorough mixing, the coupling solutions were added to the synthesis vessel containing the deprotected resin. If necessary, additional NMP was added to fully suspend the resin. Couplings were incubated for a minimum of 40 minutes at room temperature. Following each coupling, the resin was washed with ~20 mL NMP (3x, 10 min per wash). The resin was then deprotected with ~20 mL of 20% piperidine in NMP (2x, 10-20 min per treatment), and washed with ~10 mL NMP (3x, 5 min per wash). Repeated cycles of coupling and deprotection were then used to assemble the target sequence. Following coupling of the Fmoc-Ala-OH residue and removal of the Fmoc group, the peptide chain was acetylated at its N-terminus via overnight treatment with a capping solution consisting of acetic anhydride (0.94 mL, 10 mmol) and DIPEA (1.74 mL, 10 mmol) in ~6 mL of NMP. This acetylation step yielded resin bound intermediate **S2** (**Fig. S8A**). The 4-methyltrityl (Mtt) protecting group on the lysine side chain was then removed by treatment with 15 mL of a solution of 94:5:1 CH_2_Cl_2_/TIPS/TFA (3x, 5 min per treatment). The resin was not agitated during this step. The resin was next washed with ~20 mL of NMP (3x, 5 min per wash) to yield resin-bound intermediate **S3** (**Fig. S8A**). At this stage the resin was partioned into two equal portions, and half of this material was extended from the lysine ε-amine to generate the complete sequence of LPAT-LII. The coupling of these additional residues was achieved using methods analogous to those described above. Finally, Boc-2-aminobenzoic acid was coupled to the terminal leucine residue using similar procedures in order to install the 2-aminobenzoyl (Abz) fluorophore. Following completion of the synthesis, the resin was washed with NMP (3x) and CH_2_Cl_2_ (3x). A 5 mL solution of 95:2.5:2.5 TFA/TIPS/H_2_O was then used to cleave LPAT-LII from the resin (2x, 30 min per treatment). The resin was not agitated during the cleavage step. The cleaved peptide solution was concentrated on a rotary evaporator, and the remaining residue was added dropwise to 35 mL of diethyl ether chilled over dry ice. The suspension was centrifuged at 4000 rpm for 5 minutes at 4 ^o^C to collect precipitated LPAT-LII peptide. The diethyl ether was decanted and the crude peptide was dried overnight under vacuum.

Crude LPAT-LII was resuspended in a minimum volume of 1:1 MeCN/H_2_O and purified by RP-HPLC using a Dionex UltiMate 3000 HPLC system equipped with a Phenomenex Luna 5 μm, 100 Å C18 column (10 x 250 mm) [aqueous (95:5 H_2_O/MeCN, 0.1% formic acid) / MeCN (0.1% formic acid) mobile phase at 4.0 mL/min, method: hold 20% MeCN (0.0-2.0 min), linear gradient of 20-45% MeCN 2.0-7.0 min, linear gradient of 45-55% MeCN 7.0-12.0 min, linear gradient of 55-90% MeCN 12.0-13.0 min, hold 90% MeCN 13.0-14.5 min)]. Pure peptide fractions were concentrated on a rotary evaporator and lyophilized. Purified LPAT-LII peptide was then resuspended in DMSO at a final concentration of 20 mM. The identify and purity of LPAT-LII in this stock solution were confirmed by LC-ESI-MS (**Fig S8A**) and RP-HPLC (**Figure S8B**). These analyses were conducted on a Dionex UltiMate 3000 HPLC system interfaced with an Advion CMS expression^L^ mass spectrometer. Separations were achieved with a Phenomenex Kinetix® 2.6 μM C18 100 Å column (100 x 2.1 mm) [aqueous (95% H_2_O, 5% MeCN, 0.1% formic acid) / MeCN (0.1% formic acid) mobile phase at 0.3 mL/min, method: hold 10% MeCN (0.0-0.5 min), linear gradient of 10-90% MeCN (0.5-7.0 min), hold 90% MeCN (7.0-8.0 min)].

**
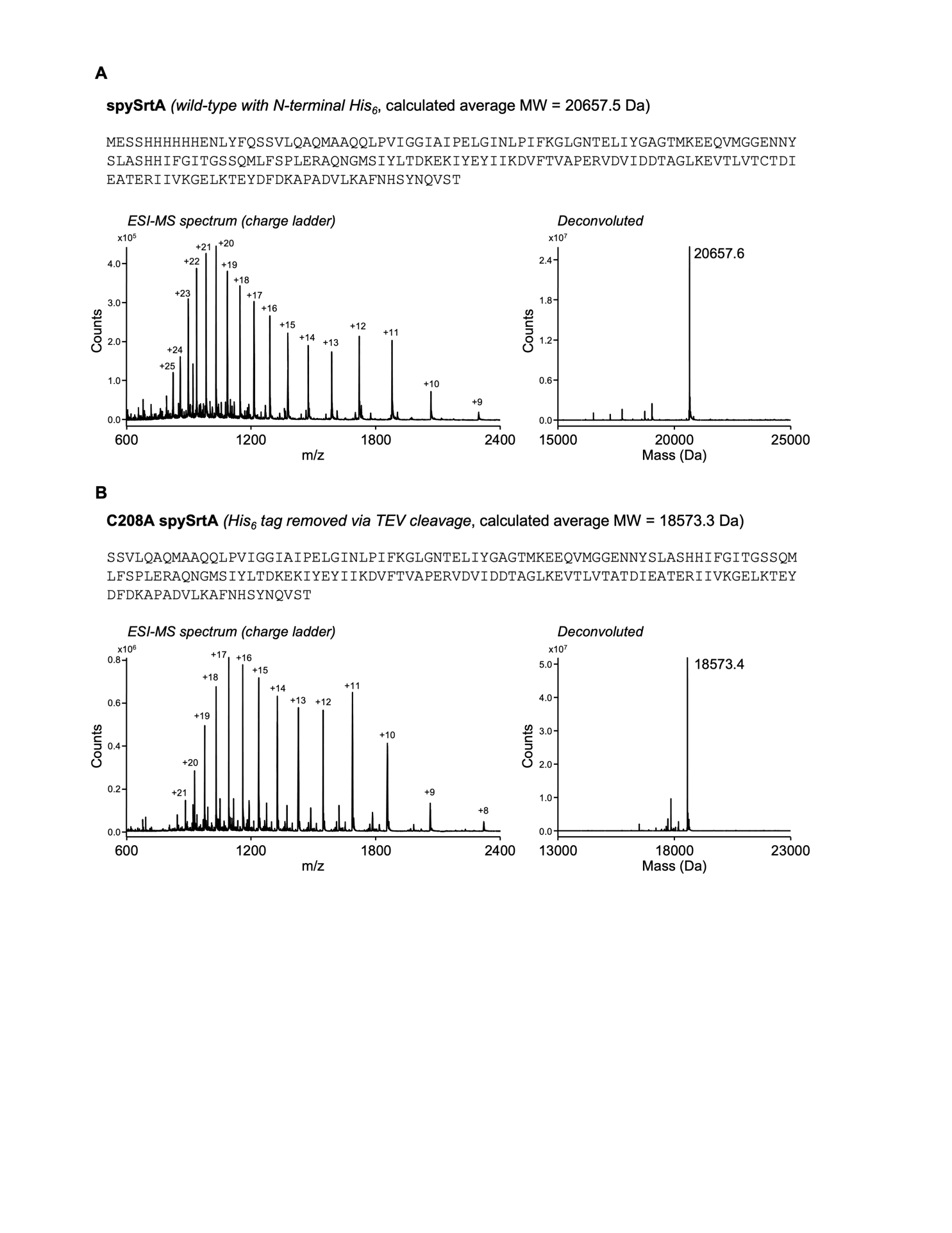
**

**Figure S1.** **Sequences and LC-ESI-MS characterization of wild-type and C208A spySrtA.** Mass spectra were acquired using an Agilent 6545XT AdvanceBio Q-TOF system as described in Materials and Methods. Unprocessed spectra showing full charge ladders for each protein are shown, along with the corresponding deconvoluted spectra.

**
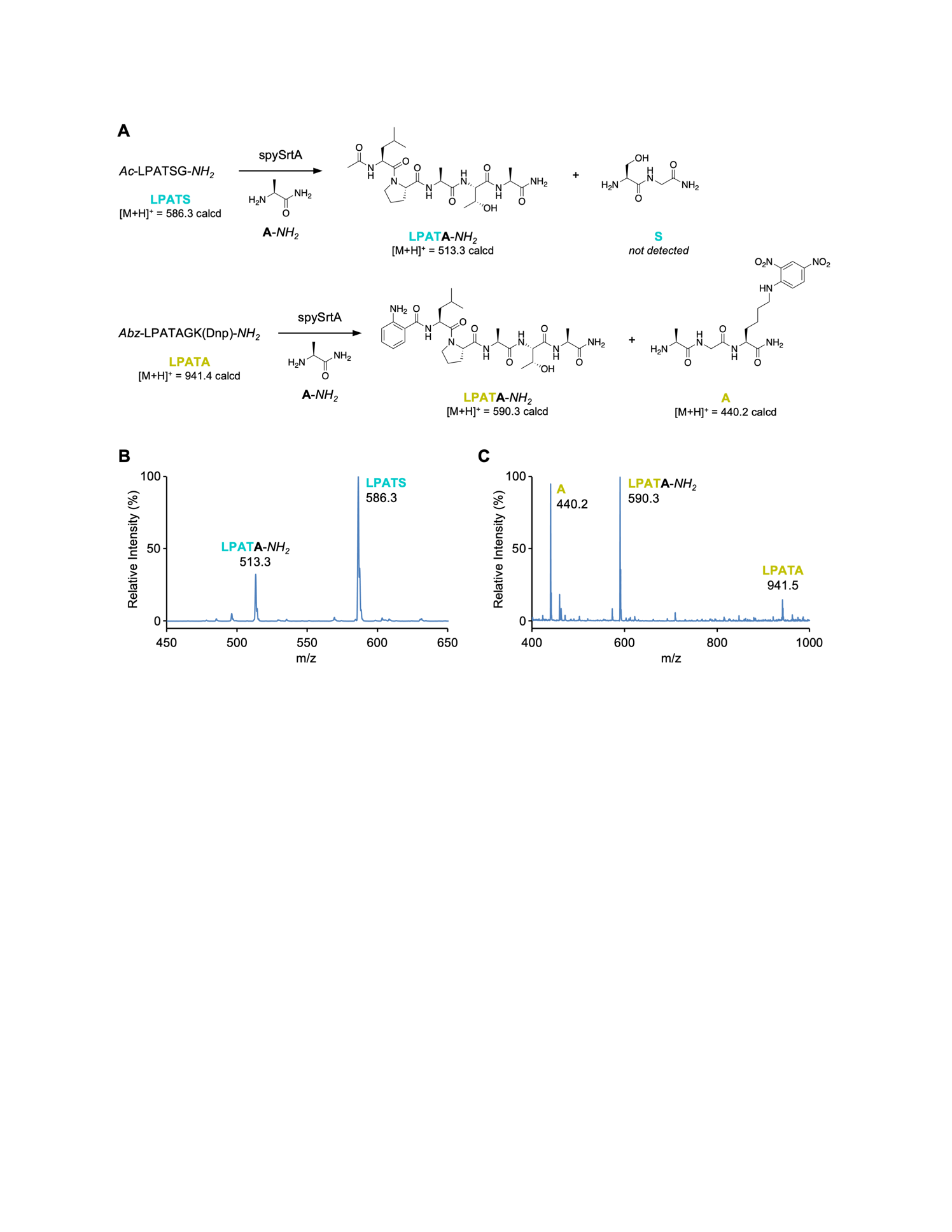
**

**Figure S2. Model transacylation reactions of spySrtA with LPATA and LPATS substrates. (A)** Schemes for the reaction of model peptides (*Ac*-LPATSG-*NH_2_* or *Abz*-LPATAGK(Dnp)-*NH_2_*) with excess alanine amide (A-*NH_2_*) in the presence of spySrtA. Conditions: 50 μM LPATS/LPATA substrate, 5 mM A-*NH_2_*, 1 or 5 μM spySrtA, 10% v/v sortase reaction buffer (500 mM Tris, 1500 mM NaCl, pH 7.5), room temperature. **(B)** ESI-MS spectrum of crude reaction mixture with *Ac*‑LPATSG-*NH_2_* (5 μM spySrtA, 180 min time point). **(C)** ESI-MS spectrum of crude reaction mixture with *Abz*-LPATAGK(Dnp)-*NH_2_* (1 μM spySrtA, 260 min time point).

**
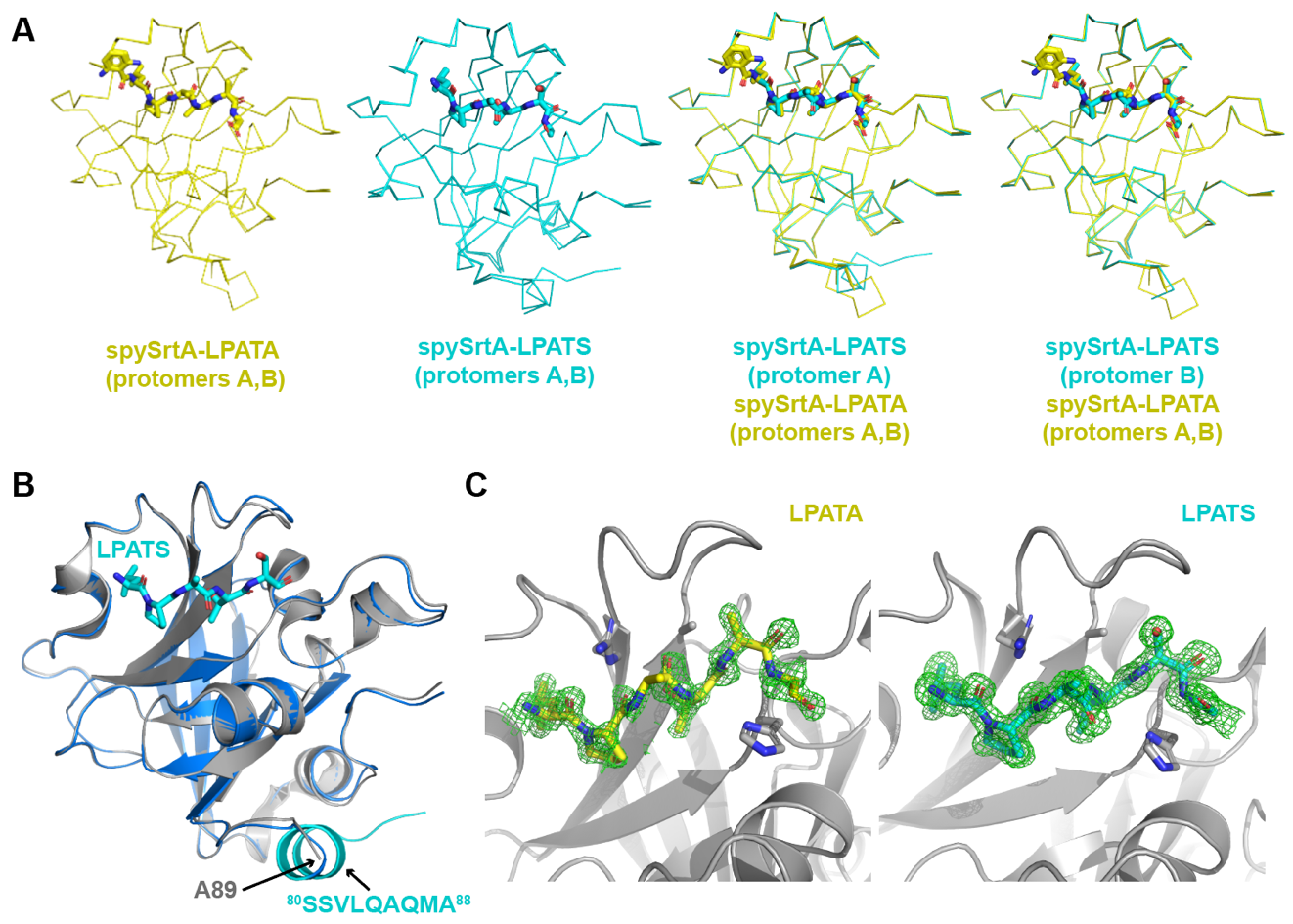
**

**Figure S3.** **Structural alignments of spySrtA complex structures. (A)** Pairwise alignments for all protomers of both structures reveal that the main-chain atoms of spySrtA-LPATS align to protomer A or protomer B of spySrtA-LPATA with RMSD = 0.124 Å (544 atoms) and 0.114 Å (556), respectively. Alignment of protomer B of spySrtA-LPATS with the two protomers of spySrtA-LPATA resulted in RMSD values of 0.126 Å (569) and 0.124 Å (574), respectively. Within each structure, the protomers align within the experimental error: RMSD = 0.075 Å (558) for spySrtA-LPATA and 0.089 Å (499) for spySrtA-LPATS. All structures are shown in ribbon representation and colored as labeled. The peptides are shown as sticks and colored by heteroatom (O = red, N = blue). **(B)** Although the apo spySrtA protein previously crystallized (PDB ID 3FN5, gray cartoon) started at residue Ser81, residues N-terminal to Ala89 are unresolved, perhaps due to the presence of an additional 18 residues including a His-tag and Thrombin cleavage site. The spySrtA protein crystallized in our spySrtA-LPATS structure (blue cartoon with the peptide shown as in **(A)**), starts at Ser80 and all residues are resolved. The N-terminal residues form an additional α-helix, as shown in cartoon, colored cyan, and labeled. **(C)** Unbiased electron density maps are shown for each of the spySrtA-LPATA and spySrtA-LPATS structures. The peptide atoms were removed and a round of refinement was run using the experimental reflections data. Protomer A of each of the complex structures is shown in gray cartoon and aligned with the refined structure. The modeled peptides are shown as sticks and colored by heteroatom, as labeled. Catalytic triad residues (H142, C208A, and R216) are in gray sticks. In each, the *F*_o_-*F*_c_ maps are rendered at 2.5σ and shown in the vicinity of the peptide.

**
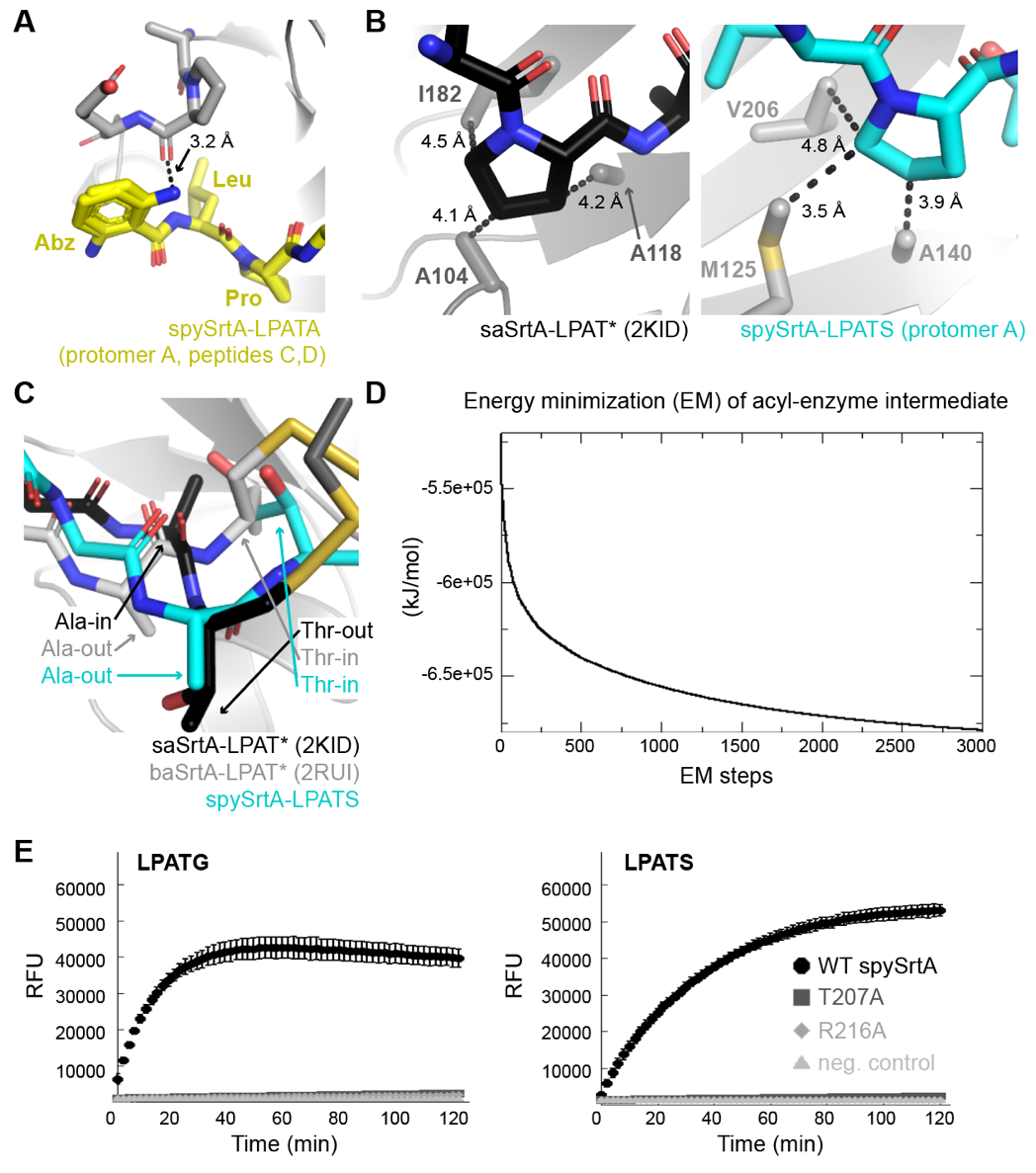
**

**Figure S4. The spySrtA LPATA- and LPATS-complex structures. (A)** The Abz moiety at the N‑terminus of the *Abz*-LPATAGK(Dnp)-*NH_2_* peptide is in two conformations in the peptides bound to protomer A (chain C) and B (chain D) in the spySrtA-LPATA structure. The peptides are in stick representation and colored by heteroatom (N=blue, O=red, C=yellow). The spySrtA protein is in gray cartoon, with the interacting residue, P188, and neighboring residues, A187 and E189, as sticks and colored by heteroatom. The distance between the carbonyl of P188 and amide of Abz is labeled and shown as a black dashed line. **(B)** A comparison of the potential interactions of the P3 Pro with SrtA enzymes, including from the saSrtA-LPAT* (PDB ID 2KID) structure (left) and spySrtA‑LPATS structure (right). The peptides are shown as stick residues and colored by heteroatom and as labeled. The spySrtA enzymes are in gray cartoon, with interacting residue side chains as sticks and colored by heteroatom (C=black/cyan, S=golden yellow). Distances are labeled and measurements shown as black dashed lines. **(C)** The peptides from the saSrtA-LPAT* (2KID), baSrtA-LPAT* (2RUI), and spySrtA‑LPATS structures are shown in stick representation and colored by heteroatom (as in (**A-B**)) to highlight the differences in orientation of the P2 Ala and P1 Thr residues. Orientation of “Ala-in/out” and “Thr-in/out” are indicated by the colored arrows. (**D**) Energy minimization of the spySrtA-LPAT acyl-enzyme intermediate model solvated in water. **(E)** Triplicate fluorescence data (in relative fluorescence units, RFU) for the reaction of *Abz*-LPATGGK(Dnp)-*NH_2_*, *left*, or *Abz*-LPATSGK(Dnp)-*NH_2_*, *right*, and H_2_NOH in the presence of WT (black circles), T207A (dark gray squares), and R216A (gray diamonds) spySrtA protein, and a no protein control (light gray triangles). The key is the same for both graphs. Data for the *Abz*-LPATAGK(Dnp)-*NH_2_* peptide is in **Fig. 4D**.

**
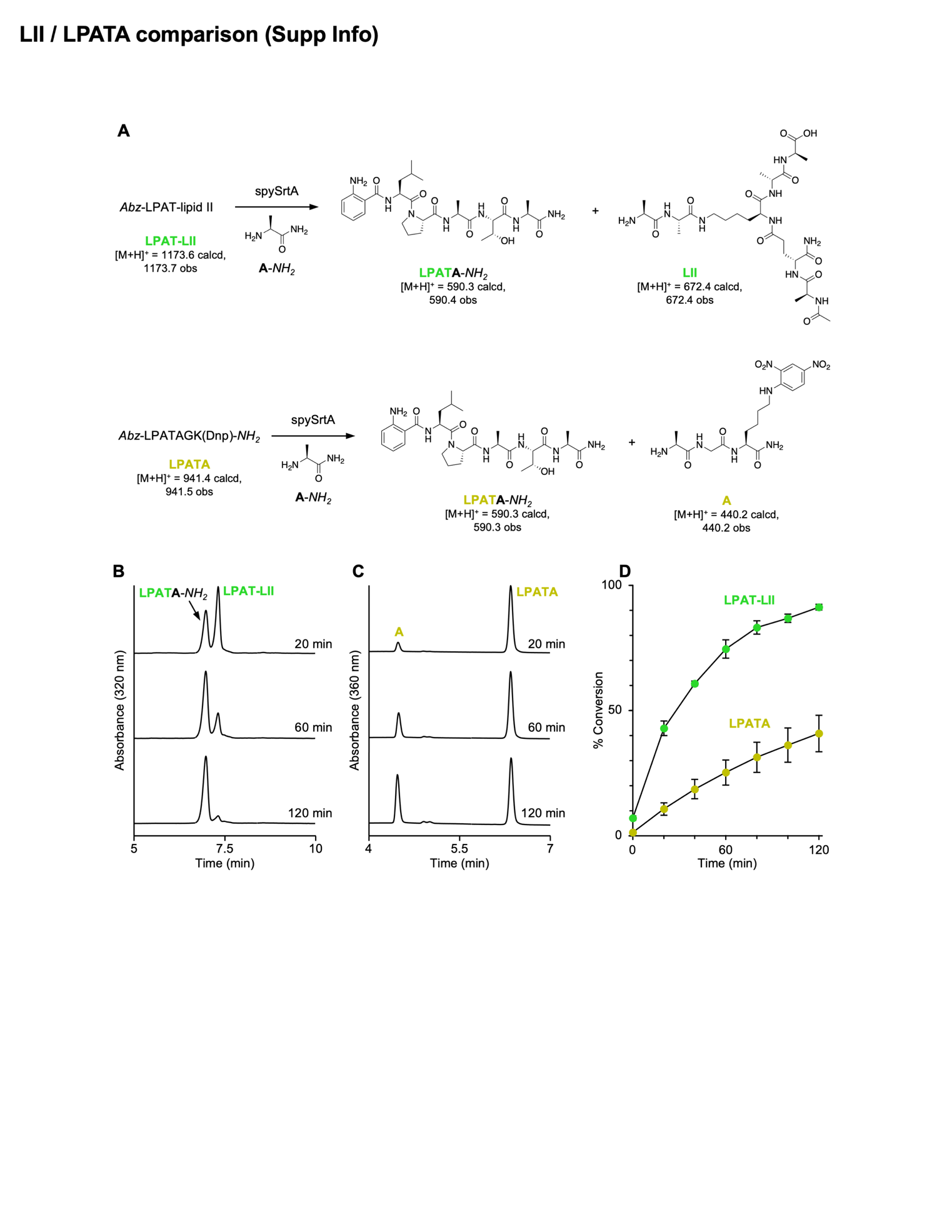
**

**Figure S5. Comparison of transacylation reactions with LPAT-LII and LPATA. (A)** Model transacylation reactions of *Abz*-LPAT-lipid II (LPAT-LII) or *Abz*-LPATAGK(Dnp)-*NH_2_* (LPATA) with excess alanine amide (A-*NH_2_*) in the presence of spySrtA. Conditions: 50 μM LPAT-LII/LPATA peptide, 5 mM A-*NH_2_*, 1 μM spySrtA, 10% v/v sortase reaction buffer (500 mM Tris, 1500 mM NaCl, pH 7.5), room temperature. Reaction progress was monitored by RP-HPLC, and the identity of all reaction components was confirmed by LC-ESI-MS. Representative RP-HPLC chromatograms for the reactions of **(B)** LPAT-LII and **(C)** LPATA are shown. **(D)** Time course of reactions in panel **(A)** demonstrating a higher level of substrate conversion for LPAT-LII as compared to LPATA. Percent conversion values were estimated by comparing RP-HPLC peak areas for the unreacted substrates and the specific reaction products labeled in panels **(B)** and **(C)**. Data points represent three independent experiments (mean ± standard deviation).

**
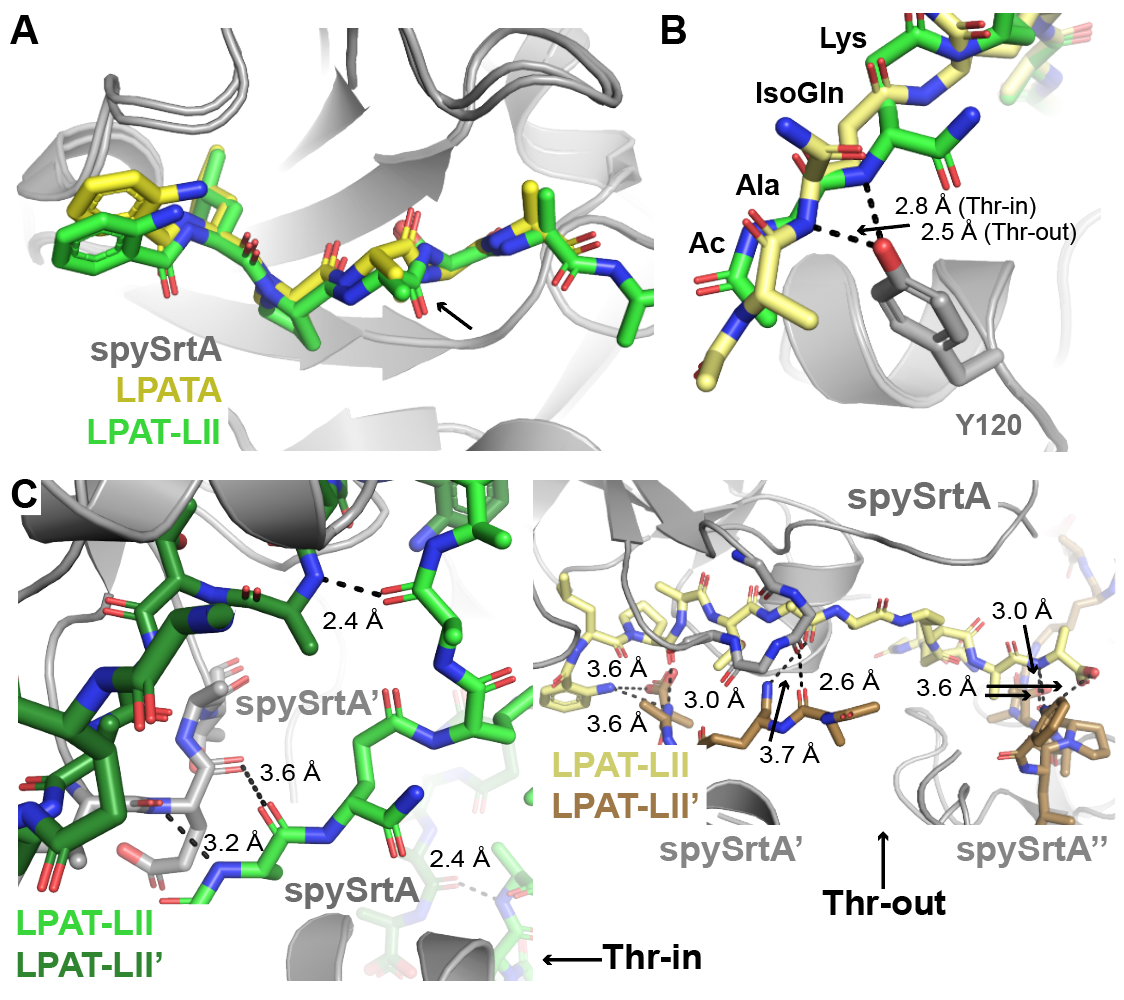
**

**Figure S6. Structure of spySrtA bound to LPAT-LII peptide.** The structures in these figures are rendered similarly: the spySrtA protein is shown as gray cartoon, with any side chains or highlighted areas as sticks and colored by heteroatom (N=blue, O=red). The LPAT-LII molecules are in green sticks and colored by heteroatom. Molecules related by symmetry are labeled, indicated by single or double apostrophes. Ligand LPAT-LII molecules related by symmetry are in dark green sticks and colored by heteroatom and labeled. Distances are shown as black dashed lines and labeled. **(A)** The similarities in the LPATA sequence of the spySrtA-LPATA and LPAT-LII bound structures are highlighted. The black arrow indicates the most significant difference, which is a rotation in the P2 Ala carbonyl. This reflects conformational flexibility seen in the P2 Ala and P1 Thr residues amongst the structures presented in this work. **(B-C)** These figures illustrate interactions of LPAT-LII with the spySrtA enzyme **(B)** and molecules, both ligand and protein, related by symmetry **(C)**. In **(B)**, both LII pentapeptides are shown and the residues are labeled. The “Thr-in” peptide (green carbons) is shifted by approximately one residue as compared to the “Thr-out” LII peptide (pale yellow carbons); however, it is the amide of the isoglutamine residue that interacts with the hydroxyl of Y120 in both structures. The “Thr-in” and “Thr-out” structures are labeled in **(C)**.

**
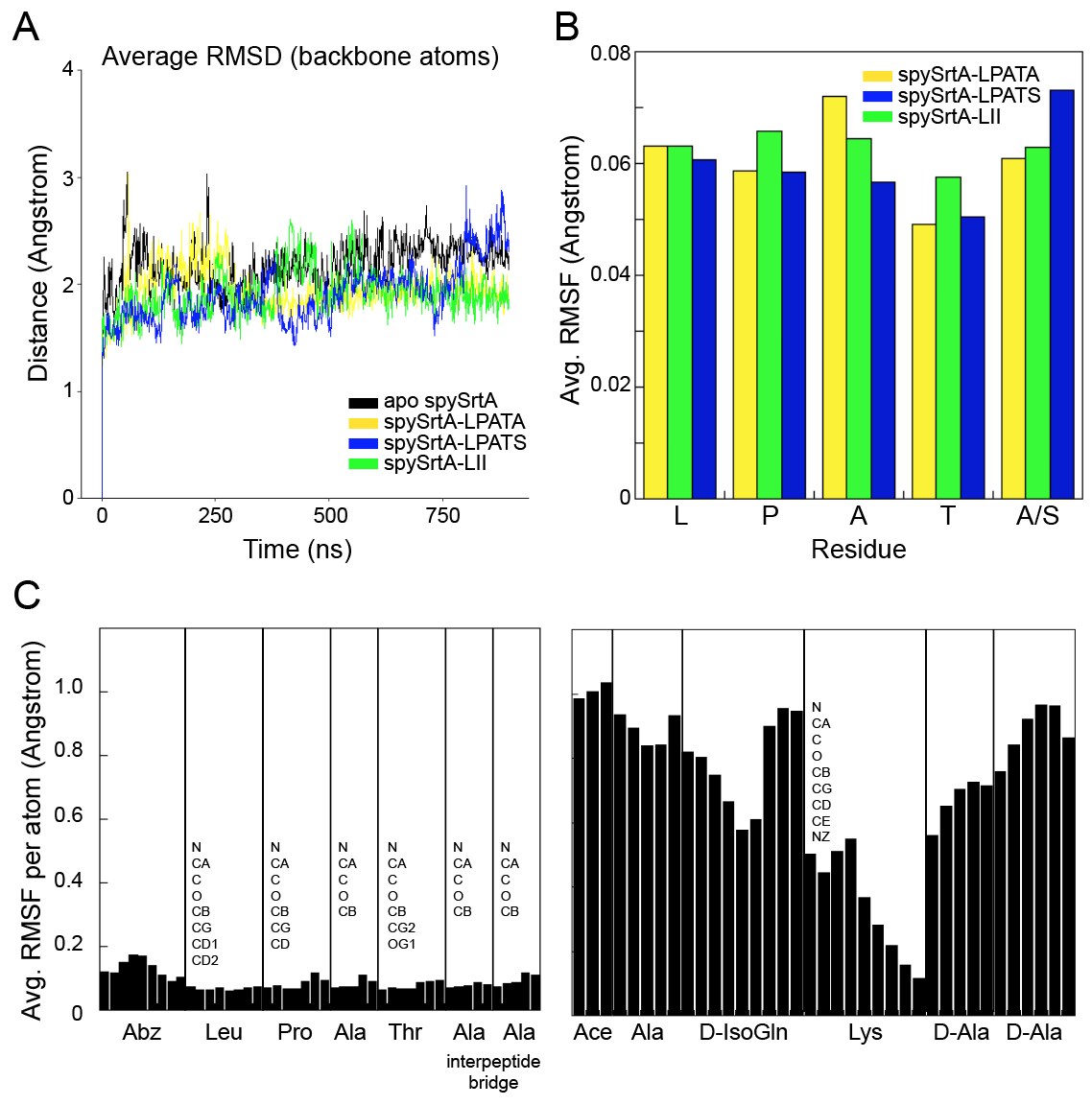
**

**Figure S7. Molecular dynamics simulations of spySrtA. (A)** The average root-mean-square-deviation (RMSD) for backbone atoms over the course of the simulation is graphed for all four simulations, including the apo spySrtA (PDB ID 3FN5, black curve) protein, as well as the structures presented here, spySrtA‑LPATA (yellow), spySrtA-LPATS (blue), and spySrtA-LPAT-LII “Thr-in” (green). **(B)** The average root-mean-square-fluctuation (RMSF) from the average position over the course of each simulation is graphed for the L-P-A-T-A/S residues, colored as in **(A)** and labeled. These values are an average of the RMSF for the backbone atoms of each residue. **(C)** The RMSF for each atom of the LPAT-LII molecule is graphed for Abz-LPATAA (left) and pentapeptide of LII (right); Ac = acetyl group. The order of atoms is backbone then side chain for all residues, and the exact order is listed for the LPATAA segment and the Lys residue of the pentapeptide, highlighting the increased flexibility for the Lys atoms starting at the Cε atom of the sidechain.


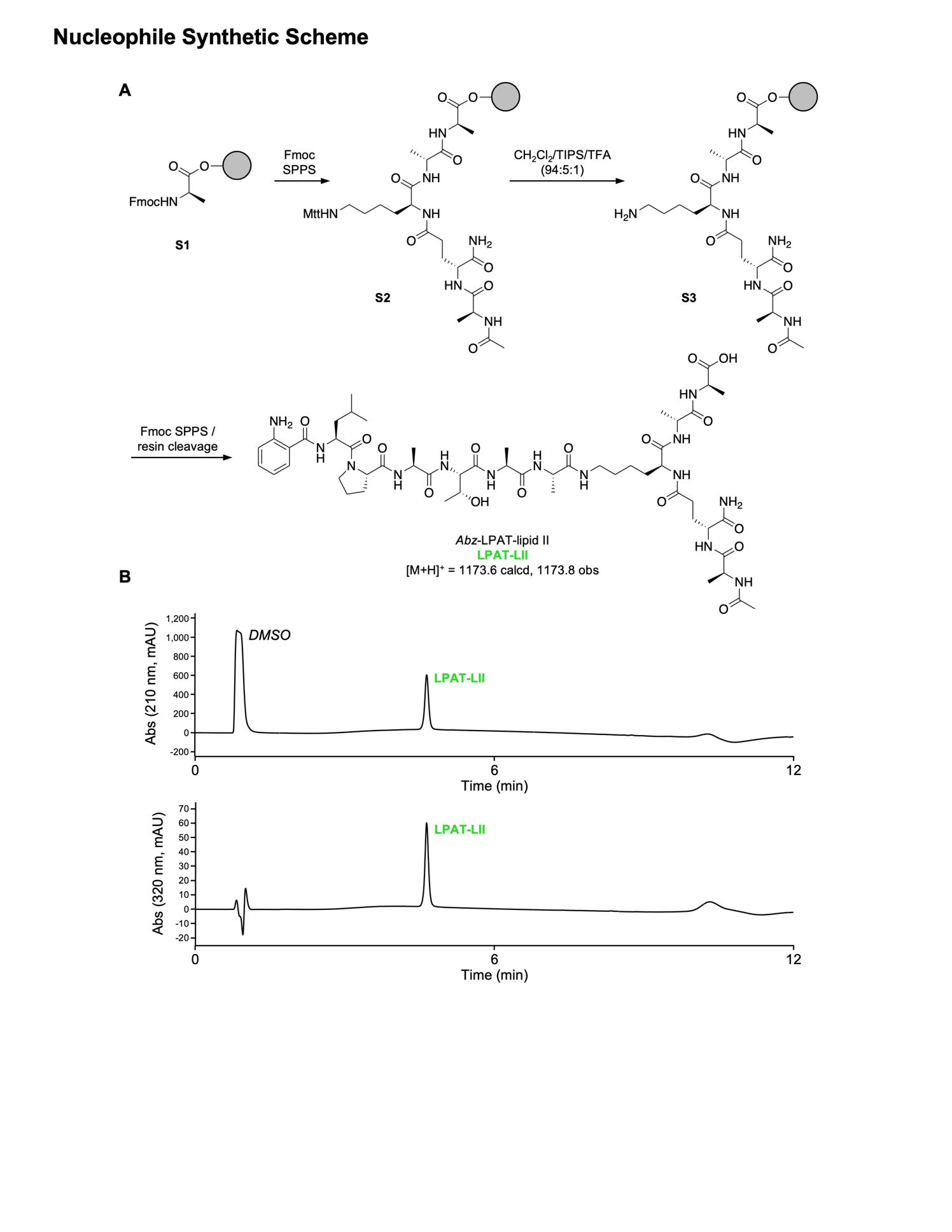


**Figure S8. Synthesis and characterization of LPAT-LII. (A)** Synthetic scheme for the solid phase syntheis of *Abz*-LPAT-lipid II (LPAT-LII). The identity of purified LPAT-LII was confirmed by LC-ESI-MS. **(B)** RP-HPLC characterization of purified LPAT-LII. The presence of a peak in the 320 nm chromatogram is consistent with the expected absorbance of the 2-aminobenzoyl (Abz) fluorophore.

**Table S1. Details of the molecular dynamics simulation size.**

| **System** | **Total number of atoms** | **Cubic box dimensions [nm]** | **Simulation time [ns]** |
| --- | --- | --- | --- |
|  |  |  |  |
| SpySrtA (Apo)^a^ | 34123 | 7.0224 | 896.6 |
|  |  |  |  |
| SpySrtA-LPATA^b^ | 34118 | 7.0186 | 905.0 |
|  |  |  |  |
| SpySrtA-LPATS^c^ | 32523 | 6.9350 | 905.0 |
|  |  |  |  |
| SpySrtA-LPAT-LII^d^ | 35836 | 7.1559 | 1005.0 |
|  |  |  |  |

^a^PDB ID 3FN5

^b^PDB ID 7S51

^c^PDB ID 7S40

^d^PDB ID 7T8Y

**Supplemental References**

1. Wang J, Wolf RM, Caldwell JW, Kollman PA, Case DA (2004) Development and testing of a general amber force field. *J Comput Chem* 25(9):1157–1174.

2. Cornell WD, Cieplak P, Bayly CI, Kollmann PA (1993) Application of RESP charges to calculate conformational energies, hydrogen bond energies, and free energies of solvation. *J Am Chem Soc* 115(21):9620–9631.

3. Lu T, Chen F (2012) Multiwfn: a multifunctional wavefunction analyzer. *J Comput Chem* 33(5):580–592.

4. Barca GMJ, et al. (2020) Recent developments in the general atomic and molecular electronic structure system. *J Chem Phys* 152(15):154102.

5. Essmann U, et al. (1995) A smooth particle mesh Ewald method. *J Chem Phys* 103(19):8577.

6. Bussi G, Donadio D, Parrinello M (2007) Canonical sampling through velocity rescaling. *J Chem Phys* 126(1):014101.

7. Parrinello M (1981) Polymorphic transitions in single crystals: A new molecular dynamics method. *J Appl Phys* 52(12):7182.

8. Hess B, Bekker H, Berendsen HJC, Fraaije JGEM (1997) LINCS: A linear constraint solver for molecular simulations. *J Comput Chem*.

9. Towns J, et al. (2014) XSEDE: accelerating scientific discovery. *Comput Sci Eng* 16(5):62–74.

10. Tribello GA, Bonomi M, Branduardi D, Camilloni C, Bussi G (2014) PLUMED 2: New feathers for an old bird. *Comput Phys Commun* 185(2):604–613.
